# Proteomic analyses of age related changes in A.BY/SnJ mouse hearts

**DOI:** 10.1186/1477-5956-11-29

**Published:** 2013-07-01

**Authors:** Krishnatej Nishtala, Truong Quoc Phong, Leif Steil, Martina Sauter, Manuela Gesell Salazar, Reinhard Kandolf, Stephan B Felix, Uwe Völker, Karin Klingel, Elke Hammer

**Affiliations:** 1 Interfakultäres Institut für Genetik und Funktionelle Genomforschung, Universitätsmedizin Greifswald, Friedrich-Ludwig-Jahn-Str. 15A, 17487 Greifswald, Germany; 2 Abteilung Molekulare Pathologie, Universitätsklinikum Tübingen, Tübingen, Germany; 3 Klinik für Innere Medizin B, Universitätsmedizin Greifswald, Greifswald, Germany

**Keywords:** Aging, Hearts, Murine model, Differential in-gel electrophoresis, LC-MS based quantitation

## Abstract

**Background:**

A.BY/SnJ mice are used to study pathological alterations in the heart due to enteroviral infections. Since age is a well-known factor influencing the susceptibility of mice to infection, response to stress and manifestation of cardiovascular diseases, the myocardial proteome of A.BY/SnJ mice aged 1 and 4 months was comparatively studied using two dimensional-differential in-gel electrophoresis (2D-DIGE) and liquid chromatography tandem mass spectrometry (LC-MS/MS).

**Results:**

Complementary analyses by 2D-DIGE and gel-free LC-MS/MS revealed 96 distinct proteins displaying age associated alterations in their levels. Proteins related to protein transport, and transport chain, lipid metabolism and fatty acid transport showed significant changes in 4 months old mouse hearts compared to juvenile hearts. Proteins involved in lipid metabolism and transport were identified at significantly higher levels in older mice and dysregulation of proteins of the respiratory transport chain were observed.

**Conclusion:**

The current proteomics study discloses age dependent changes occurring in the hearts already in young mice of the strain A.BY/SnJ. Besides alterations in protein transport, we provide evidence that a decrease of ATP synthase in murine hearts starts already in the first months of life, leading to well-known low expression levels manifested in old mice thereby raising the possibility of reduced energy supply. In the first few months of murine life this seems to be compensated by an increased lipid metabolism. The functional alterations described should be considered during experimental setups in disease related studies.

## Background

Aging is one of the risk factors in the incidence and progression of cardiovascular diseases like congestive heart failure, myocardial infarction (MI), arteriosclerosis, and hypertension [1-4]. Over the life time functional and structural changes such as increased thickness of the ventricular wall, increased cavity size, and myocardial stiffness occur in the heart [5]. Such age related changes do not only occur in humans but also in rodents and dogs which are often used as animal models to investigate pathomechanisms of cardiovascular diseases. Thus, a decline in mitochondrial function, decreased oxidative phosphorylation, and increased oxidative modification of proteins have been reported as a function of aging [6,7], as well as the presence of post-translational modifications of proteins [8]. It is evident from this knowledge that processes related to aging cannot be neglected in studies targeting the molecular changes occurring in heart diseases.

Murine models of coxsackievirus B3 (CVB3)-induced myocarditis are widely used to study mechanisms leading to chronic myocarditis and dilated cardiomyopathy (DCM) since they perfectly mimic the human disease patterns [9]. Age related aspects are important in such models in two ways: On one hand, the age of the host plays an important role in susceptibility to the infecting CVB3 virus and thereby disease development [10]. On the other hand, chronic viral myocarditis and the subsequent development of DCM with cardiac remodeling progresses in susceptible mouse strains over a period of time, up to months. Thus, pathogenic mechanisms were studied in chronic CVB3 myocarditis up to 120 days post infection (p.i.) in susceptible A/J mice [11]. In our earlier studies of enteroviral myocarditis and DCM in A.BY/SnJ mice, we reported changes in the heart proteome due to CVB3 infection at different stages of the disease starting from the initial stages of infection to chronic stages at 84 days p.i [12,13]. It is obvious that mice also age during this period and hence, it is important to use age matched controls in such disease studies, but also to know the magnitude of alterations in such controls. Aging has been studied in mammals using traditional proteomics techniques of 2-DE and mass spectrometry disclosing changes in post translational modification of proteins [7] as well as in the level of proteins in different subcellular fractions [8] as well as at global scale [14,15]. However, earlier proteomic studies in hearts have focused at later stages of aging mostly 6 months and beyond. Grant et al. have studied the effects of aging in the left ventricle of rats comparing 4 months old hearts with 26 months old hearts [16]. In mouse models, mice of the strain CB6F1 were screened for age-related changes between 3 months, 15 months and 23 months [14]. Using advanced quantitation techniques like SILAC, around 4000 proteins have been quantified in different tissues including heart in C57BL/6JN mice in an age range from in 5 months to 23 months [17]. However, the study reported here is the first that addresses changes in the protein profile of 4 months old mouse hearts compared to juvenile 1 month old hearts in A.BY/SnJ mice using complementary techniques of 2-D DIGE and gel-free LC-MS/MS analysis. In our analysis, we observed changes in intracellular protein/vesicular transport, lipid metabolism and mitochondrial respiratory chain.

## Results

### Proteomic profiling of age related changes in A.BY/SnJ mice by 2-D DIGE

**Figure 1:**
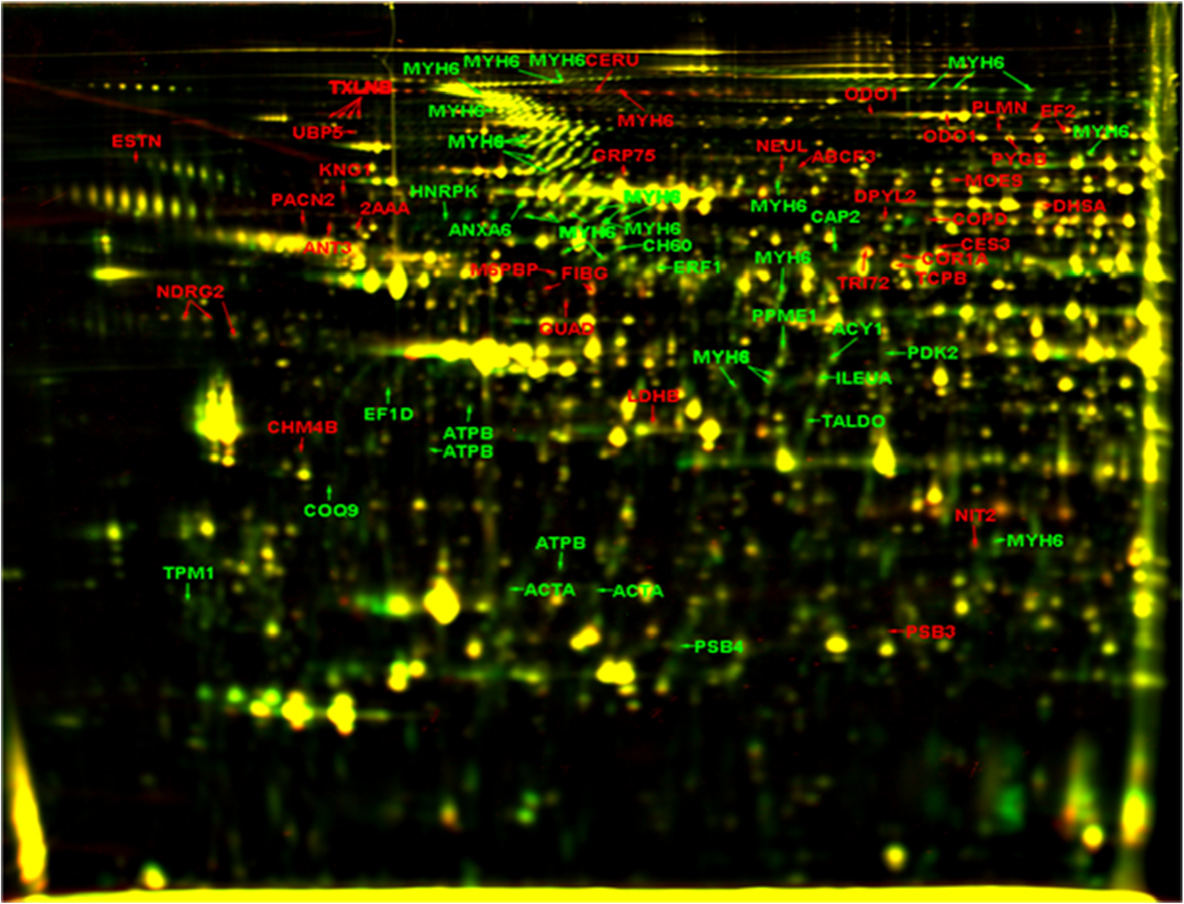
**Changes in the protein profile of mouse hearts due to aging.** Representative dual channel overlay of mice hearts 1 month old and 4 months old in the pH range of 4–7. A total of 1683 spots were detected on the gel using Delta 2D software (Decodon). Statistical analysis with Genespring (Agilent) revealed 176 spots with significantly altered intensities (*p* ≤ 0.05) of which 86 spots were identified by mass spectrometry. 40 (58) spots labeled in red showed ≥ 1.5 fold higher intensities, whereas 46 (118) spots labeled in green displayed > 1.5 fold lower intensities in the 4 months old mouse hearts compared to 1 month controls. Proteins which did not change in intensity appear in yellow.

We applied a 2-D DIGE approach to characterize the changes occurring in the hearts of A.BY/SnJ mice due to aging between the first and fourth month of life. A representative overlay picture of the protein pattern is shown in Figure 1. Image analysis using Delta-2D software resulted in detection of 1683 spots on the gel of which 176 spots passing the statistical cut of *p* ≤ 0.05 showed an intensity fold change of ≥ 1.5. Out of these, 58 spots displayed higher intensities in the range of 1.5 to 5.0 fold in hearts of older animals and 118 spots displayed lower intensities in the same hearts ranging from −1.5 to −15.8 fold.

For 86 spots excised from silver stained gels corresponding proteins were identified by LC-MS/MS analysis which revealed 50 distinct proteins (Additional file 1: Table S1). Eight proteins were identified in multiple spots suggesting protein isoforms or possible post-translational modifications since most of these spots were observed in the same molecular weight range suggesting the existence of different protein species [18]. Elongation factor 2 (EF-2), beta-taxilin (TXLNB), fibrinogen gamma (FIBG), protein NDRG2 (NDRG2), 2-oxoglutarate dehydrogenase (ODO1), actin, aortic smooth muscle isoform (ACTA), mitochondrial ATP synthase ß subunit (ATPB) and myosin-6 (MYH6) were identified in more than one spot. Myosin-6 (MYH6) was observed to be distributed over the entire gel suggesting a large number of truncated products as supporting our previous analyses [12]. Particularly, mitochondrial ATP synthase ß subunit (ATPB) was identified in multiple spots on the lower end of the gel suggesting possible degradation/truncation [13].

### Complementary analysis of age related changes in protein profile by gel-free LC-MS/MS approach

Using 2-D DIGE we were able to resolve and identify proteins in the pI range of 4–7. A particular strength of this approach is its ability to identify proteins species. To increase the protein coverage and include also membrane proteins in the analysis, we employed a LC-MS/MS approach. A total of 357 proteins were identified by LTQ-Orbitrap XL analysis with at least 2 unique peptides per protein. Among these proteins, 134 had isoelectric points outside the pI range of 4–7, as were 94 proteins belonging to either cell, mitochondrial, endoplasmic or cytoplasmic membrane (Additional file 2: Table S2) which were not covered by DIGE analysis due to limited pI range or the poor resolution of proteins with transmembrane domains on 2D gels. Of the 357 proteins represented in the MS data sets, those covered with ≥ 6 spectra per protein in at least one of the two conditions were considered for quantitation by spectral counting which is not affected by the presence of detergents like CHAPS which was used during protein extraction. 58 proteins which passed a two-tailed Students *t*-test with *p* ≤ 0.1 and displayed a ratio fold difference of 1.2 were considered to display different levels in hearts of 1 and 4 months old mice (Additional file 3: Table S3). An additional analysis of 6 months old mice (n = 4) supported age dependent differences in level for at least 36 proteins (Additional file 3: Table S3).

### Comprehensive analysis of 2-D DIGE and LC-MS/MS approaches

**Figure 2:**
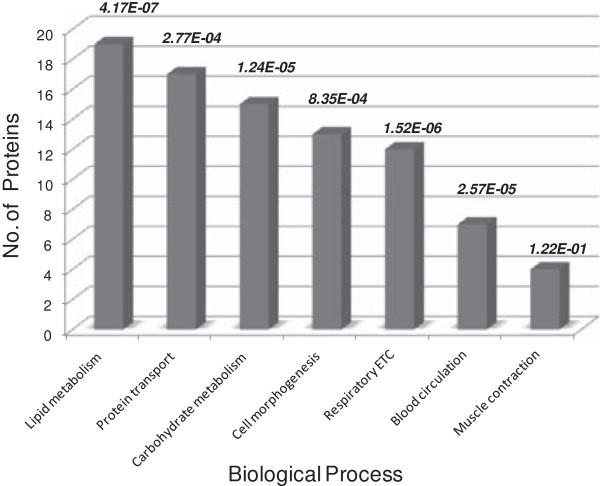
**Biological processes affected during aging in 4 months old mice heart in comparison to 1 month old animals.** Results from gel-free LC-MS/MS and 2-D DIGE analysis were analyzed together for enrichment of biological processes using PANTHER. The most significantly affected pathways are represented in the graph in the order of number of proteins classified per protein class. The corresponding *p*-values were represented at the top of each column.

To interpret the results of the two proteomics approaches from a functional perspective, proteins displaying differential levels in either the 2-D DIGE (48) or gel-free LC-MS/MS (58) analysis were submitted to the PANTHER software suite (http://www.pantherdb.org). This analysis revealed that proteins related to the functional classes protein transport (17), lipid metabolism (19), carbohydrate metabolism (15), cell morphogenesis (13), respiratory electron transport chain (12) displayed significant alterations in level during aging (Figure 2, Table 1).

**Table 1:** Protein classes significantly altered during aging of A.BY/SnJ mice*

**Functional class**	**Acc. No**	**Protein name**	**2D-DIGE**	**LC-MS/MS**
Protein transport	Q8VBT1	Beta-taxilin	3.67	n.d.
	Q8VCM7	Fibrinogen gamma chain	3.63	n.d.
	Q91YP2	Neurolysin, mitochondrial	1.85	n.d.
	Q1XH17	Tripartite motif-containing protein 72	1.68	0.91
	O89053	Coronin-1A	1.60	n.d.
	P61979	Heterogeneous nuclear ribonucleoprotein K	0.06	0.39
	Q01853	Transitional endoplasmic reticulum ATPase		1.41
	Q541Z9	Rab GDP dissociation inhibitor beta		3.86
	Q8BGK2	[Protein ADP-ribosylarginine] hydrolase-like protein 1		3.54
Lipid metabolism	Q80XN0	D-beta-hydroxybutyrate dehydrogenase, mitochondrial		12.20
	Q924X2	Carnitine O-palmitoyltransferase 1, muscle isoform		1.87
	Q99NB1	Acetyl-coenzyme A synthetase 2-like, mitochondrial		1.64
	P52825	Carnitine O-palmitoyltransferase 2, mitochondrial		1.58
	P47934	Carnitine O-acetyltransferase		1.48
Respiratory ETC./Energy metabolism	Q9CQJ8	NADH dehydrogenase [ubiquinone] 1 beta subcomplex subunit 9		2.35
	Q9DC69	NADH dehydrogenase [ubiquinone] 1 alpha subcomplex subunit 9, mitochondrial		2.05
	Q9DCS9	NADH dehydrogenase [ubiquinone] 1 beta subcomplex subunit 10		1.56
	Q61941	NAD(P) transhydrogenase, mitochondrial		1.43
	Q03265	ATP synthase subunit alpha, mitochondrial		0.83
	Q9D3D9	ATP synthase subunit delta, mitochondrial		0.82
	P62897	Cytochrome c, somatic		0.64
	P56392	Cytochrome c oxidase polypeptide 7A1, mitochondrial		0.41
	Q9CQC7	NADH dehydrogenase [ubiquinone] 1 beta subcomplex subunit 4		0.36
	Q8K2B3	Succinate dehydrogenase [ubiquinone] flavoprotein subunit, mitochondrial	2.93	1.11
	Q60597	2-oxoglutarate dehydrogenase E1 component, mitochondrial	1.82/1.51	1.39

* p-values and coefficients of variation are provided in Additional file 3: Table S3.

**Figure 3:**
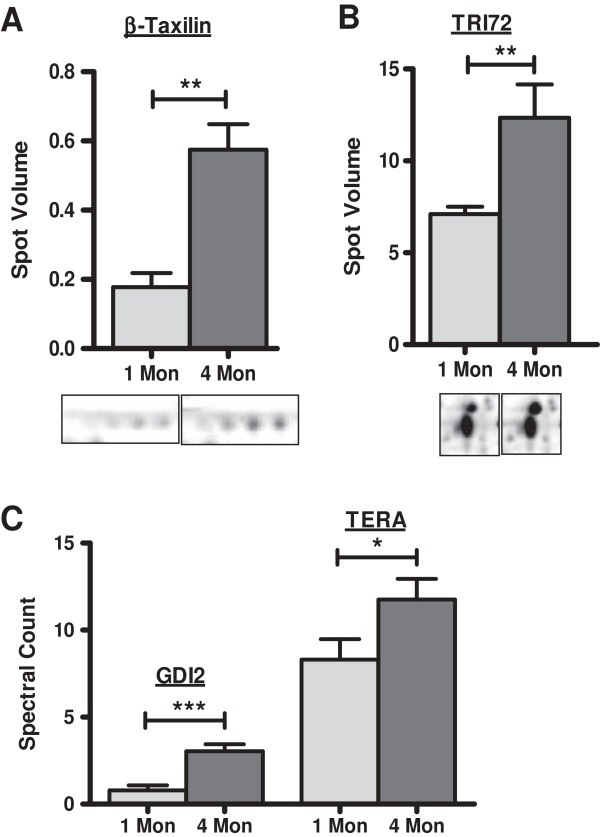
**Increase in intracellular transport proteins identified by DIGE and gel-free LC-MS/MS analysis.** Proteins involved in intracellular transport were identified in higher amounts in 4 months old mouse hearts compared to 1 month old hearts. Gray bars: 1 Month mice hearts, dark bars: 4 months mice hearts. **A**- Protein ß-taxilin (TXLNB) was identified in multiple spots and in higher amounts in 4 months hearts compared to control mice. **B**- Tripartite motif-72 (TRI72) showed approx. 1.7 fold increase in aging mice hearts. **C**- Proteins Rab GDP dissocation inhibitor 2 (GDI2) and Transitional endoplasmic reticulum ATPase (TERA) were identified in increasing amounts in aging mice hearts by gel-free LC-MS/MS analysis. * indicates *p* ≤ 0.1 ** indicates *p* ≤ 0.05 and *** indicates *p* ≤ 0.01.

Intracellular transport proteins such as beta-taxilin (TXLNB), Rab GDP dissociation inhibitor beta (GDIB), and [Protein ADP-ribosylarginine] hydrolase-like protein 1 (ARHL1) displayed higher amounts in hearts of 4 months old animals compared to those of 1 month old mice. Furthermore, tripartite motif 72 (TRI72), coronin-1A (COR1A), transitional endoplasmic reticulum ATPase (TERA)/valosin containing protein which are also related to protein transport showed significantly higher levels in older mice making intracellular protein transport the most significantly altered protein class affected by aging in our analysis (Figure 3).

**Figure 4:**
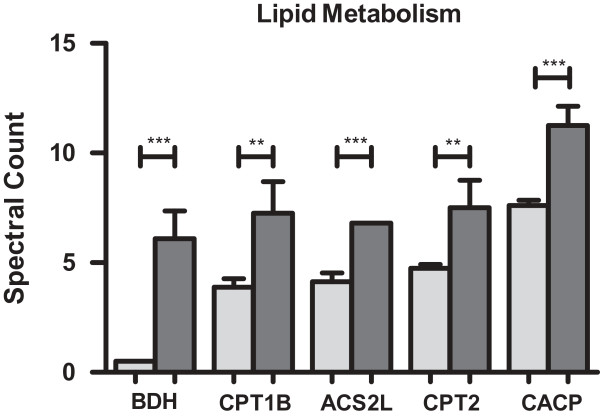
**Changes in lipid metabolism proteins in aging hearts by gel-free LC-MS/MS analysis based on spectral counts.** High levels of proteins involved in lipid metabolism were observed in hearts of 4 months old mice in comparison to those of 1 month old animals suggesting increased energy production by lipid metabolism. Protein abundance in hearts of 1 month old mice: (grey bars), of 4 months old mice (dark bars). BDH-D-beta-hydroxybutyrate dehydrogenase, mitochondrial, CPT1B-carnitine O-palmitoyltransferase 1, muscle isoform, ACS2L-acetyl-coenzyme A synthetase 2-like, mitochondrial, CPT2-carnitine O-palmitoyltransferase 2, mitochondrial, CACP-carnitine O-acetyltransferase. ** indicates p ≤ 0.05 and *** indicates p ≤ 0.01.

Proteins belonging to inner or outer mitochondrial membrane and mitochondrial matrix proteins involved in lipid metabolism and respiratory electron transport chain (ETC.) also showed differences in their levels between both groups of mice. Thus, carnitine-O-palmitoyltransferase 1, muscle isoform (CPT1B), mitochondrial carnitine-O-palmitoyltransferase 2 (CPT2), carnitine-O-acetyltransferase (CACP), involved in fatty acid transport as well as mitochondrial D-beta-hydroxybutyrate dehydrogenase (BDH), mitochondrial acetyl-coenzyme A synthetase 2-like (ACS2L) which are important in lipid metabolism were identified in higher amounts in hearts of 4 months old mice compared to those of 1 month old animals (Figure 4).

**Figure 5:**
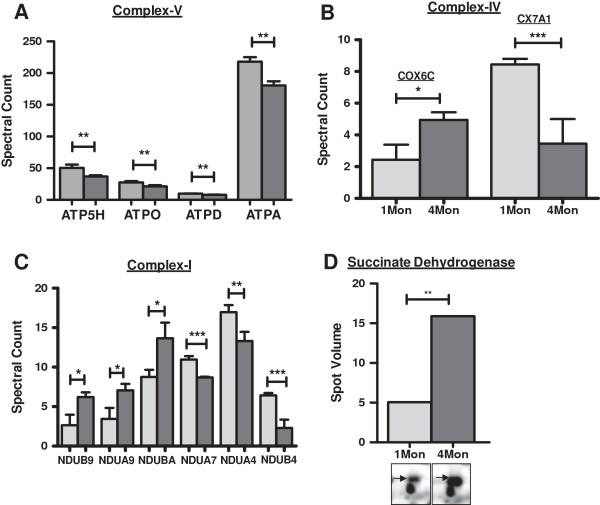
**Age dependent changes in abundance of proteins of the electron transport chain in mouse hearts.** Different subunits of respiratory electron transport chain were affected due to aging in mice hearts. Grey bars: level in hearts of 1 month old animals, dark bars: level in hearts of 4 months old mice. **A**- subunits of ATP synthase (complex V) identified by gel-free analysis - ATP5H- ATP synthase subunit d, mitochondrial, ATPO- ATP synthase subunit O, mitochondrial, ATPD- ATP synthase subunit delta, mitochondrial, ATPA- ATP synthase subunit alpha, mitochondrial. **B**- cytochrome c oxidase subunit 6C (COX6C) and polypeptide 7A1, mitochondrial (CX7A1). **C**- subunits of complex I: NADH dehydrogenase [ubiquinone] 1 beta subcomplex subunit 9 (NDUB9), NADH dehydrogenase [ubiquinone] 1 alpha subcomplex subunit 9, mitochondrial (NDUA9), NADH dehydrogenase [ubiquinone] 1 beta subcomplex subunit 10 (NDUBA), NADH dehydrogenase [ubiquinone] 1 alpha subcomplex subunit 7 (NDUA7), NADH dehydrogenase [ubiquinone] 1 alpha subcomplex subunit 4 (NDUA4), NADH dehydrogenase [ubiquinone] 1 beta subcomplex subunit 4 (NDUBA). **D**- succinate dehydrogenase (2D DIGE results) of electron transport chain. **p-value ≤ 0.05, *** indicates p ≤ 0.01 and.*indicates p ≤ 0.1.

Alterations were also observed for proteins of the respiratory electron transport chain. Different subunits of mitochondrial complex V-ATP synthase subunit alpha (ATPA), ATP synthase subunit delta (ATPD), ATP synthase subunit O (ATPO), ATP synthase subunit d (ATP5H) - were observed in lower amounts in 4 months old mice compared to juvenile ones except for ATP synthase subunit b (AT5F1) which was identified in higher amounts. Mitochondrial ATP synthase beta, (ATPB) was also observed in lower amounts in 2-D DIGE analysis in two spots. However, based on previous observations we assume that these spots constitute truncated products since they were identified in a lower molecular weight region on the gel than expected [12]. In contrast, succinate dehydrogenase (DHSA) of complex II was identified at higher amounts by 2-D DIGE analysis (Figure 5). Similar results were also observed for complex I and cytochrome c oxidase of complex IV by LC-MS/MS analysis where 3 subunits of mitochondrial NADH dehydrogenase (ubiquinone) 1 (NDUA9, NDUB9 and NDUBA) were identified in higher amounts whereas other subunits of the same enzyme (NDUA4, NDUA7 and NDUB4) were identified in lower amounts in 4 months old than in juvenile mice. Mitochondrial cytochrome c oxidase polypeptide 7A1, (CX7A1) of complex IV was identified at lower amounts in 4 months old mice whereas cytochrome c oxidase subunit 6c was identified at higher amounts. The reason for such contrasting outcomes in a group of proteins with similar function is not clear. It remains to be elucidated if the differences observed represent normal variation of subunit constitution which is compensated by the cell or if this might really lead to radical formation.

Besides the functional classes mentioned above, some proteins (4) involved in muscle contraction such as cardiac muscle troponin T (TNNT2) and transgelin (TAGL) were observed in lower amounts by gel-free LC-MS/MS analysis in juvenile mice whereas 2-D DIGE analysis showed lower amounts of protein species of tropomyosin 1 alpha chain (TPM1) and myosin-6 (MYH6) in this group compared to 4 month old mice. Furthermore, increased levels of stress protein 70 (GRP75) were observed in 4 months old A.BY/SnJ mice.

### Validation of proteins displaying age dependent changes in A.BY/SnJ mice hearts

**Figure 6:**
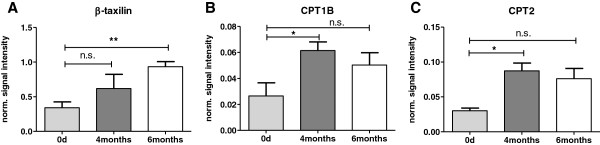
**Validation of age dependent changes in the level of proteins by immune blotting. A**-**C**: Western Blotting results: Quantitative analysis of 4 bioreplicates of hearts from 1, 4, and 6 months old mice using the software ImageQuant (GE HealthCare Life Sciences, Munich, Germany) with normalization of signal intensities to GAPDH as loading control. CPT1B: carnitine O-palmitoyltransferase 1, muscle isoform; CPT2: carnitine O-palmitoyltransferase 2. Levels of significance are indicated: * p < 0.05; ** p < 0.01. The data confirm the alterations in the myocardial protein pattern observed by 2D DIGE and LC-MS/MS analysis in 4 months old A.BY/SnJ mice hearts compared to juvenile mouse hearts.

In order to confirm the relevance of the findings by gel based and gel free proteomic analyses, candidates of different functional classes were selected for validation by Western blot analysis and immunohistochemical staining. Western blot analysis confirmed the increasing levels in mice hearts in an age range from 1 to 6 month for the transport protein ß-taxilin, muscle isoform of carnitine O-palmitoyltransferase 1, and carnitine O-palmitoyltransferase 2 (Figure 6A-C, Additional file 4: Figure S1) Immunohistochemical analyses confirmed that heterogeneous nuclear ribonucleoprotein K (HNRPK) involved in RNA processing displayed decreased levels whereas Rab GDP dissociation inhibitor beta (GDIB) and stress protein 70 (GRP75) involved in intracellular protein transport were identified at higher levels in hearts of 4 months old mice compared to juvenile animals (Additional file 5: Figure S2). Thus, the expression differences of these six proteins in cardiac tissue between 1 and 4 months old animals observed by 2D DIGE and LC-MS/MS approach were confirmed.

## Discussion

Aging is one of the many factors that influence the susceptibility of the heart to diseases including congestive heart failure, coronary heart disease, atherosclerosis, myocardial infarction and hypertension. Lakatta et al. have shown that the chances of developing arteriosclerosis increase significantly with age, and differences in cardiac remodeling due to aging have also been reported [1-3,19]. Most or all of the diseases mentioned above develop over long periods paralleled by age progression. Under such circumstances, it is essential to use age matched controls to dissect the effects of aging from disease mediated changes. By employing the complementary methods of 2-D DIGE and gel-free LC-MS/MS, we studied the differences in the myocardial protein pattern of 1 and 4 months old mice.

In general, the alterations observed were moderate in terms of number of altered proteins and fold changes which is in line with the findings reported for hearts of aged C57BL/6mice [17]. Of particular interest were the increased levels of proteins involved in protein transport/vesicular transport in hearts of 4 months old mice compared to hearts of 1 month old animals. Among them, ß-taxilin (TXLNB) was identified in multiple protein species, which showed approximately three fold increase in intensity in 2-D DIGE analysis. ß-taxilin is known to be abundantly expressed in skeletal muscle and heart and is involved in the transport of vesicles to the plasma membrane. It is considered to be the human homologue of chicken MDP77 for which increased expression was also observed during development [20-22].

An increase of vesicular transport in aging was also indicated by higher levels of tripartite motif-containing protein 72 (TRI72) also named mitsugmin 53 (MG53). Overexpression of this skeletal and cardiac muscle specific protein enhanced vesicular trafficking to the sarcolemmal membrane and its knock-out impeded myoblast differentiation in mice [23-25]. Besides the trafficking proteins, also a regulator of such transport processes, Rab GDP dissociation inhibitor beta (GDIB) showed elevated levels in hearts of 4 months old mice. Due to the association of the Rab proteins with secretory and endocytic pathways, these proteins play an important role in regulation of vesicular transport and help in docking of transport vesicles with their corresponding acceptor membranes [26]. In this context, transitional endoplasmic reticulum ATPase (TERA)/Valosin-containing protein as an ATP binding protein is thought to influence the protein-protein interactions in membrane transport by its association with clathrin - a structural protein involved in receptor-mediated endocytosis [27], also of the cardiotropic CVB3 [28]. Increase in the levels of proteins involved in protein/vesicular transport can be inferred as a secondary effect leading to endoplasmic reticulum associated protein degradation (ERAD) by either the proteasome or macroautophagy pathway. Also, we have observed an increase in intensity of stress protein 70 (GRP75) involved in the protein recognition step of ERAD [29].

Other pathways which were significantly influenced in the hearts of the 4 months old animals were lipid metabolism and ß-oxidation. Notably, increased levels were observed for three different proteins involved in fatty acid transport CPT1B, CPT2, and CACP. CPT1 and CPT2 are reported to be involved in the translocation of long chain fatty acids located in the inner mitochondrial membrane into the matrix. CPT1B is known to be abundantly expressed in the heart and is thought to be activated by long chain fatty acids via PPARα. In mammals, continuous circulation of lipids is under the influence of CPT1 gene transcription. In PPARα knockout mice, a decreased expression of muscle-CPT1 was observed which effects main energy production. PPARα is known to regulate myocardial lipid metabolism and controls transcription of muscular CPT1 [30]. Besides CPT1 and CPT2, higher levels of carnitine acetyl transferase were observed in hearts of 4 months old mice. Although the carnitine level was not measured in this study, this finding might be interpreted as a compensatory effect, because decreased levels of cardiolipin and impaired pyruvate metabolism were reported in aged rats [31]. In turn, administration of acetyl-L-carnitine to old rats (28 months) restored the metabolic functions such as pyruvate oxidation and transport compared to young rats (5 months) [32]. Other mitochondrial enzymes such as long chain acyl CoA dehydrogenase, very long chain acyl CoA dehydrogenase, short chain acyl CoA dehydronenase involved in fatty acid metabolism were also identified in the current analysis, but their levels in the heart did not display any age-related alterations. Therefore, taking all data together, an increased fatty acid turn over can be supposed which might reflect the higher energy demands of 4 months old mouse hearts.

The highest number of altered proteins in the heart was assigned to the functional category respiratory electron transport chain/oxidative phosphorylation. Individual subunits of complexes I to IV showed minor changes in their levels. For example, subunits of complex I, such as NDUA9, NDUB9 and NDUBA were identified in higher amounts in hearts of 4 months old mice whereas NDUA4, NDUA7 and NDUB4 were identified in lower amounts pointing towards disproportion in the complex I composition during early stages of growth. These findings are in agreement with reports on ambiguous ratios of complex I subunits in LVs of aged rats identified by iTRAQ [16]. Furthermore, similar results were obtained by investigating age related differences in mice regarding the mRNA level of complexes I-IV. NADH subunit 1, cytochrome c oxidase subunit 3 and cytochrome b were observed in reduced levels in old hearts (20 months) compared to young hearts (4 months) in C57BL/6 mice [33]. This indicates that oxidative phosphorylation is affected during aging and that capacity for ATP synthesis might be reduced in older animals. This hypothesis is supported by our data, which revealed differences for subunits of complex V/ATP synthase (ATP5H, ATPO, ATPD, and ATPA) which were identified in lower amounts in hearts of 4 months old mice. Our observations are in line with those reported by Chakravarti et al. [15] in hearts of aged mice (age of 24 months).

Using complementary 2-D DIGE and gel-free LC-MS/MS analysis, our study is the first focusing on proteome alterations in mouse hearts during the first 4 months of life, a time span which is often studied in respect to the development of viral heart disease in different mouse models (A.BY/SnJ, ASW, C57BL/6). Our findings are in line with those of earlier reports of aging in mice and rat hearts which focussed on later time points (up to 20 months and older). Apparently, similar pathways/reactions display aging related changes both in young and older animals (see also influence on oxidative phosphorylation mentioned above). The pathways influenced in our study are also comparable to those reported by Grant et al. [16], where hearts of young 4 months rats were compared to 26 months old animals using an iTRAQ based quantitative approach. Obviously, for a set of proteins (mitochondrial pyruvate dehydrogenase E1 component subunit beta, phosphoglycerate kinase 1, hemoglobins HBA and HBB, desmin, troponin T2, tropomyosin alpha 1, cytochrome c, and voltage dependent anion channel-1) a decrease of amount can be observed over the life time in different rodents. In C57BL/6 mice lower levels of transcripts were observed for cytochrome c and tropomyosin alpha 1 in older animals (4 → 20 months), too [33].

Summarizing our 2-D DIGE and those from Dai et al. [14], where protein levels in the heart of CB6F1 mice of 3, 15 and 23 months were analyzed, a positive correlation with aging seems to exist for the levels of protein species of translation elongation factor 2 and myoglobin. A summary of data from our study and the literature (Table 2) shows, that at least some protein alterations known to be manifested in the heart during aging starts already in quite young animals.

**Table 2:** Aging related alterations in rodents

**Pathway/Protein**	**Gene**	**This study**		**Literature findings**			
		1 → 4 m*	**method**	**4 → 24 m**^**# **^**rodent method**			**Ref.**^**§**^
***Electron transport chain***							
ATP synthase subunit d.	Atp5h	**down**	**LC-MS**				
ATP synthase subunit O	Atp5o	**down**	**LC-MS**				
ATP synthase subunit delta.	Atp5d	**down**	**LC-MS**				
ATP synthase subunit alpha	Atp5a1	**down**	**LC-MS**	down	mouse	2DE	[15]
ATP synthase subunit beta	Atp5b	**down**	**2D DIGE**	down	mouse	2DE	[15]
cytochrome c	Cycs	**down**	**LC-MS**	down	mouse	mRNA	[33]
***metabolism***							
mito. pyruvate dehydrogenase E1 component subunit beta	Pdhb	**down**	**LC-MS**	down	rat	itraq	[16]
phosphoglycerate kinase 1	Pgk1	**down**	**LC-MS**	down	rat	itraq	[16]
***stress-related proteins***							
heat shock protein 70	Hspa9	**up**	**2D DIGE**	down	mouse	mRNA	[33]
60 kDa heat shock protein	Hspd1	**down**	**LC-MS**				
***others***							
desmin	Desm	**down**	**LC-MS**	down	mouse	2DE	[15]
troponin T2	Tnnt2	**down**	**LC-MS**	down	rat	itraq	[16]
tropomyosin alpha 1	Tpm1	**down**	**2D DIGE**	down	rat, mouse	itraq, mRNA	[16,33]
voltage dependent anion channel-1	Vdac1	**down**	**LC-MS**	down	rat	itraq	[16]
elongation factor2	Eef2	**up**	**2D DIGE**	up	mouse	2DE	[14]

* Differences between hearts of 1 month and 4 months old animals obtained within this study in A.BY/SnJ mice. Column content in bold letters.

^#^ Differences between hearts of ≥ 4 months and ≥ 20 months old mice published by other authors.

^§^*Ref.* reference.

However, such life spanning continuous correlation certainly does not apply to all proteins. An example of the latter class could be stress protein 70, which is involved in proper protein folding. Hsp70 was found to be expressed at higher levels in hearts of 4 months old compared to 1 month old mice in our study (Table 2). However, decreased hsp70 levels were frequently found in old animals and seem to be related to a less efficient protein repair in older mammals in case of stress. Another example might be Mn- and Cu-Zn superoxide dismutases, which were both identified in our analysis (Additional file 2: Table S2), but did not display age-dependent differences in levels. This observation might indicate onset of oxidative stress at later stages of aging post 4 months since changes of levels of these proteins were more prominent between 15 months and 23 months [14].

## Conclusions

Using complementary 2D-DIGE and gel-free LC-MS/MS analysis proved advantageous in gaining a comprehensive view on the differences in the myocardial protein pattern in 1 and 4 months old mice. Our analyses reveal that protein/vesicular transport, lipid metabolism and mitochondrial ETC. which play an important role in the survival and energy production of cells are already displaying alterations in levels in the early stages of life in A.BY/SnJ mice which are often used as animal model to study viral heart diseases. The alterations observed in the protein patterns might have an influence on the susceptibility towards disease and might support the understanding of the pathological changes. Our results show the magnitude of alterations occurring in juvenile mice which have to be considered in study design when A.BY/SnJ mice are used as a model.

## Materials and methods

### Mouse heart tissue

A.BY/SnJ mice (H-2b) were kept under specific pathogen-free conditions at the animal facilities of the Department of Molecular Pathology, University Hospital Tübingen. Experiments were conducted according to the German animal protection law and permission was obtained by a governmental committee (Permit No. PA2/10) on animal welfare as well as an Institutional Animal Care and Use Committee. Mice were sacrificed after 1, 4, or 6 months (n = 4) and collected hearts were immediately snap frozen in liquid nitrogen.

### Sample preparation

Hearts were homogenized into fine powder with 150 μl of rehydration buffer (RB) containing urea 8 M, thiourea 2 M and 2% CHAPS using a Mikro dismembrator (Braun, Melsungen, Germany) at 2600 rpm for 2 min. Around 1.5 to 2 ml of RB was used for reconstitution of the tissue powder. Samples were subjected to sonication on ice three times for 5 s each with nine cycles at 80% energy using a Sonoplus (Bandelin, Berlin, Germany). Cell debris was removed by centrifugation of the tissue homogenates at 16,000 × *g* for 1 h at 4 °C. Supernatants were collected and protein concentrations were determined with a Bradford Assay using bovine serum albumin as standard protein (Pierce, Thermo Scientific, Bonn, Germany).

### Two dimensional-differential in gel electrophoresis (2D- DIGE)

Quantified protein extracts were minimally labeled with CyDyes according to manufactures’ suggestions (GE Healthcare, Munich, Germany) before electrophoresis. Individual heart protein extracts, 50 μg each (n = 4 per group) were labeled with 400 pmol of either Cy3 or Cy5 dyes. As an internal standard, aliquots of all individual samples were pooled and labeled with Cy2. The labeling of the samples was done by dye swapping such that protein extracts from two animals per group were labeled with Cy3 and Cy5 each.

Linear pH 4–7, 24 cm IPG strips (GE Healthcare) were rehydrated overnight in rehydration solution (urea 8 M, thiourea 2 M, pharmalyte 3–10 and 10 × dithiothreitol (DTT)) containing two labeled samples (Cy3 and Cy5, each 50 μg) and the corresponding internal standard (Cy2, 50 μg) for first dimension separation. The isoelectric focusing (IEF) was performed using a Multiphor II apparatus (GE Healthcare) with voltages ranging from 500 V to 3500 V for 17.50 h as suggested by the manufacturer. After IEF, the strips were reduced and alkylated in equilibration buffers containing 10% DTT w/v or 25% 2-iodoacetamide w/v, respectively, along with urea 8 M, Tris–HCl 1.5 M, pH8.8, glycerol 87% w/v, and SDS 20% w/v before second dimension separation of proteins on 12.5% SDS-polyacrylamide gels in low fluorescent glass plates [34].

### Image analysis and statistical tests

After separation in the second dimension, the gel images were recorded on a Typhoon 9400 Scanner (GE Healthcare) and analyzed with Delta 2D software version 4.0 (Decodon, Greifswald, Germany) [12]. After matching the gels, the spot volumes from Delta 2D were imported into GeneSpring GX version 7.3.1 (Agilent Technologies, Waldbronn, Germany) for analysis. Statistically significant differences in spot intensities among the groups consisting of four individual biological replicates each were calculated by one-way ANOVA applying Welch *t*-test with a *p*-value cutoff of ≤0.05.

### In-gel digestion of proteins and mass spectrometric analysis

Three preparative gels were run each with 300 μg of the pooled samples. Protein spots were visualized by silver staining as described in [35], images were recorded and aligned with those of analytical gels in Delta2D. The protein spots displaying significant changes were excised from the preparative gels and destained with potassium hexacyanoferrat. After dehydration with 100% acetonitrile, the gel spots were subjected to proteolysis with trypsin (Promega, Madison, WI, U.S.A.) overnight (16 h) at 37 °C. The tryptic peptides were sequentially extracted from the gel pieces by ultra-sonication using initially 0.1% acetic acid in 50% acetonitrile and then 0.05% acetic acid in 80% acetonitrile. The extracted peptides were separated on an Acclaim PepMap 100 reverse phase column (3 μm, 75 μm i.d × 150 mm, LC Packings, Dionex, Idstein, Germany) with a nano-HPLC (EASY-nLC, Proxeon Biosystems A/S, Odense, Denmark) coupled with LTQ-Orbitrap-XL mass spectrometer (Thermo Electron, Bremen, Germany) using a 35 min linear gradient ranging from 5-60% ACN in 0.1% acetic acid (0 min −5%B −3 min-5% -23 min-35% -28 min-60% -30 min-100% -32 min-100% -35 min-0%) at a constant flow rate of 0.3 μL/min.

### Gel-free/In-solution protein digestion and mass spectrometric analysis

Protein samples, 2 μg each, (four individual biological replicates per group), were reduced with 25 mM DTT, alkylated with 100 mM iodoacetamide before digestion with Lys C (Sigma) in the ratio of 1:100 for 3 h followed by overnight (16 h) proteolysis with trypsin (Promega) in the ratio of 1:10. After stopping the digestion with 1% acetic acid, the samples were purified on C-18 resin tips with a binding capacity of 2 μg (OMIX, Varian, Pittcon, U.S.A.). The purified peptides were separated on an Acclaim PepMap 100 reverse phase column with an EASY-nLC (Proxeon Biosystems) using a 300 min non-linear gradient ranging from 0-100% ACN in 0.1% acetic acid (0 min-0%B −15 min-0% -27 min 5-30% -290 min −60%-291 min-100% -295 min-100% -300 min-0%) at a constant flow rate of 0.3 μL/min. The separated peptides were monitored with a LTQ-Orbitrap-XL mass spectrometer (Thermo Electron).

### Identification of proteins

For the identification of proteins from 2D spots, a SEQUEST (Bioworks 3.2/SEQUEST 2.7, Thermo Electron) search was done for the resultant MS/MS spectra against Swissprot mouse database release 57.1 with a mass tolerance of 10 ppm for the precursor ions (Additional file 6: Table S4) and 1 Da for fragment ions.

Identification of proteins from gel-free LC-MS/MS analysis was performed via automated SORCERER/SEQUEST search (Sorcerer built 4.04, Sage-N Research Inc., Milpitas, CA, U.S.A.) against a Swissprot mouse forward-reverse database release 57.1 with a mass tolerance of 10 ppm (1 Da for fragment ions). Proteins identified with at least two peptides throughout the whole dataset at a Peptide Prophet probability of 90% (Additional file 6: Table S4) were later on included in quantitation by spectral counting [36].

### Quantitation by spectral counting

The resulting output files from SORCERER search were loaded into SCAFFOLD software version 2.6 (Proteome Software, Portland, U.S.A.) for analysis of spectral data. Homologous peptides shared among different proteins were removed from the dataset in order to include only protein specific peptides in protein quantitation. The data were normalized across all samples and categories. Proteins with ≥ six assigned spectra per condition were analyzed for differences by Student’s *t*-test and proteins passing *p*-value ≤ 0.1 were considered as differentially [36]. Since proteins observed with more than 30 spectra do not display linear increase of spectral counts with increasing amount [37] changes of proteins were considered as biologically relevant when fold change was > 1.2. Data were confirmed by comparison with results of hearts from 6 months old mice analyzed as described above (Additional file 3: Table S3).

### Western blotting analysis

Immuno blotting with specific antibodies was carried using a conventional semi-dry blotting method as described earlier [12] with the following primary antibodies diluted in 3% bovine serum albumine: polyclonal anti-mouse carnitine O-palmitoyltransferase 1, muscle isoform (CPT1B, rabbit, dilution 1:1000, Biozol, Eching, Germany), polyclonal anti-mouse carnitine O-palmitoyltransferase 2 (CPT2, rabbit, 1:1000, Sigma, Munich, Germany), monoclonal anti-mouse ß-taxilin (goat, 1:200, Santa Cruz Biotechnology, Inc. Dallas, TX, USA), monoclonal anti-GAPDH (rabbit, 1:1000, Cell Signaling Technology (Danvers, MA, U.S.A.). As secondary antibodies horseradish peroxidase conjugated ones from goat (anti-rabbit IgG, dilution 1:2500, Pierce, Thermo Scientific, Bonn, Germany) and rabbit (anti-goat IgG, 1:5000, Life Technologies, Darmstadt, Germany) were used. For detection, the membranes were incubated with the SuperSignal West Femto Maximum Sensitivity Substrate (Pierce, Thermo Scientific, Bonn, Germany) for 5 min, before signals were detected with a ChemoCam Imager (Intas, Göttingen, Germany). Band intensities were quantified using the ImageQuant software version 5.0 (GE Healthcare, Munich, Germany).

### Immunohistological staining

Paraffin tissue sections of 5 μm size from mouse hearts were incubated with primary antibodies of heterogeneous nuclear ribonucleoprotein K (Bethyl Laboratories, Texas, U.S.A.), heat shock protein 70 (Cell Signalling/New England Biolabs GmbH, Frankfurt am Main, Germany), and Rho GDP dissociation inhibitor 2 (Novus Biologicals, Cambridge, U.K.) for 1 h at room temperature. Then, sections were heated in 10 mM citrate buffer (pH 6.0) for 4 min at 120°C for antigen retrieval. The sections were incubated with biotinylated secondary antibodies (Vector Laboratories, Inc., Burlingame, CA, U.S.A.) for 30 min at room temperature, followed by a streptavidin-biotin-immunoperoxidase system (Vectastain Elite StreptABC, Vector Laboratories) and 3,3 diaminobenzidine (Dako, Glostrup, Denmark) or HistoGreen (Linaris, Wertheim, Germany) as substrate. Slides were counterstained with hematoxylin. Species-matched IgG was used as negative controls. The slides were viewed under a Zeiss Axioskop 40 microscope (Carl Zeiss MicroImaging, GmbH, Göttingen, Germany).

## Competing interests

The authors declare that they have no competing interests.

## Authors’ contributions

KN and TQP carried out experimental work regarding tissue disruption, protein extraction, gel based and gel free protein analysis, using mouse heart tissues provided by KK and MS who also performed IHC analysis. LS participated in the DIGE experiments and gel bioinformatic analyses. Analysis of spectral counting data was supported by MGS. RK and SBF helped in the study design and revised the manuscript. EH and KN did functional interpretation and drafted the manuscript. UV conceived and coordinated the project and revised the manuscript. All authors read and approved the final manuscript.

## Authors’ information

Co-last authors: Karin Klingel and Elke Hammer.

## Supplementary Material

Additional file 1: Table S1 Proteins identified from 2D gel spots displaying altered intensity in 4 months old mouse hearts compared to 1 month old controls. Click here for file

Additional file 2: Table S2 List of protein identified with 2 peptide confidence by LC-MS/MS analysis. Click here for file

Additional file 3: Table S3 List of proteins showing altered levels in hearts of 4 and 6 months old mice compared to 1 month old mice detected by LC-MS/MS approach. Click here for file

Additional file 4: Figure S1 Validation of age dependent changes in the level of proteins by immunohistochemical staining. Click here for file

Additional file 5: Figure S2 Western Blot analysis results of ß-taxilin, CPT1B and CPT2. Click here for file

Additional file 6: Table S4 Description of experiment settings for LC-MS/MS analysis and presentation of protein identification. Click here for file
