# Supplementary material for: Proteomic analyses of age related changes in A.BY/SnJ mouse hearts
Source: Proteome Sci. 2013 Jul 1;11:29. doi: 10.1186/1477-5956-11-29 (PMC3704963; doi:10.1186/1477-5956-11-29)
Supplement: Additional file 1: Table S1 — Proteins identified from 2D gel spots displaying altered intensity in 4 months old mouse hearts compared to 1 month old controls. [file 1477-5956-11-29-S1.pdf]

## Table S1-Proteins identified from 2D gel spots displaying altered intensity in 4 months old mouse hearts compared to controls

| Swissprot ID* | Acc no.* | Protein Annotation | Spot-ID | Fold Change | Peptide Count | p-Value | Protein Score | Sequence Coverage (%) | CV 1 Month | CV 4 Months | CV* |
|---|---|---|---|---|---|---|---|---|---|---|---|
| **Proteins altered by ≥ 1.5 fold difference due to aging in mice at 4 months** | | | | | | | | | | | |
| EF2_MOUSE | P58252 | Elongation factor 2 | ID106235 | 5.32 | 30 | 0.01 | 298.26 | 25.6 | 57.85 | 15.84 | 69.95 |
| ANT3_MOUSE | P32261 | Antithrombin-III | ID106524 | 4.04 | 10 | 0.02 | 100.21 | 27.7 | 56.33 | 24.29 | 69.34 |
| TXLNB_MOUSE | Q8VBT1 | Beta-taxilin | ID106252 | 3.67 | 17 | 0.02 | 170.22 | 22.3 | 47.37 | 6.42 | 58.01 |
| FIBG_MOUSE | Q8VCM7 | Fibrinogen gamma chain | ID106629 | 3.63 | 10 | 0.00 | 100.25 | 24.3 | 30.04 | 11.67 | 63.68 |
| PACN2_MOUSE | Q9WVE8 | Protein kinase C and casein kinase substrate in neurons protein 2 | ID374568 | 3.57 | 5 | 0.04 | 50.16 | 10.5 | 67.93 | 42.40 | 82.88 |
| EF2_MOUSE | P58252 | Elongation factor 2 | ID106234 | 3.55 | 14 | 0.01 | 140.20 | 16.3 | 38.72 | 9.11 | 55.23 |
| TXLNB_MOUSE | Q8VBT1 | Beta-taxilin | ID106251 | 3.52 | 18 | 0.04 | 180.25 | 18 | 54.79 | 10.37 | 62.45 |
| TXLNB_MOUSE | Q8VBT1 | Beta-taxilin | ID106250 | 3.32 | 11 | 0.02 | 110.25 | 22.3 | 43.52 | 16.35 | 65.56 |
| MYH6_MOUSE | Q02566 | Myosin-6 | ID106157 | 3.11 | 16 | 0.00 | 160.26 | 8.8 | 9.51 | 5.12 | 51.16 |
| DHSA_MOUSE | Q8K2B3 | Succinate dehydrogenase [ubiquinone] flavoprotein subunit, mitochondrial | ID106429 | 2.93 | 11 | 0.00 | 110.23 | 16.6 | 6.76 | 22.42 | 57.11 |
| PLMN_MOUSE | P20918 | Plasminogen | ID612408 | 2.45 | 15 | 0.00 | 150.27 | 19.8 | 26.54 | 17.08 | 49.38 |
| FIBG_MOUSE | Q8VCM7 | Fibrinogen gamma chain | ID106613 | 2.42 | 16 | 0.00 | 160.27 | 40.6 | 9.53 | 13.46 | 44.20 |
| TXLNB_MOUSE | Q8VBT1 | Beta-taxilin | ID106253 | 2.34 | 19 | 0.02 | 190.23 | 21.9 | 31.98 | 6.88 | 42.40 |
| KNG1_MOUSE | O08677 | Kininogen-1 | ID106457 | 2.18 | 7 | 0.02 | 70.22 | 10.7 | 31.41 | 21.73 | 44.68 |
| NIT2_MOUSE | Q9JHW2 | Nitrilase homolog 2 | ID106905 | 2.06 | 6 | 0.01 | 60.23 | 29 | 23.58 | 9.37 | 35.38 |
| DPYL2_MOUSE | O08553 | Dihydropyrimidinase-related protein 2 | ID106504 | 1.98 | 10 | 0.04 | 100.23 | 20.3 | 31.85 | 26.83 | 43.83 |
| TCPB_MOUSE | P80314 | T-complex protein 1 subunit beta | ID106568 | 1.97 | 9 | 0.00 | 90.25 | 19.6 | 6.56 | 5.25 | 32.43 |
| CES3_MOUSE | Q8VCT4 | Carboxylesterase 3 | ID310987 | 1.95 | 4 | 0.05 | 40.17 | 7.6 | 6.22 | 39.62 | 53.15 |
| M6PBP_MOUSE | Q9DBG5 | Mannose-6-phosphate receptor-binding protein 1 | ID106596 | 1.95 | 10 | 0.04 | 100.23 | 17.6 | 32.33 | 16.78 | 36.05 |
| CERU_MOUSE | Q61147 | Ceruloplasmin | ID106156 | 1.92 | 18 | 0.01 | 180.21 | 14.1 | 16.38 | 21.98 | 38.22 |
| ESTN_MOUSE | P23953 | Liver carboxylesterase N | ID106359 | 1.87 | 6 | 0.01 | 60.19 | 12.8 | 17.64 | 17.40 | 36.85 |
| 2AAA_MOUSE | Q76MZ3 | Serine/threonine-protein phosphatase 2A 65 kDa regulatory subunit A alpha isoform | ID106526 | 1.86 | 10 | 0.01 | 100.28 | 16.8 | 21.94 | 10.01 | 35.93 |

| | | | | | | | | | | | |
|---|---|---|---|---|---|---|---|---|---|---|---|
| NEUL_MOUSE | Q91YP2 | Neurolysin, mitochondrial | ID307182 | 1.85 | 7 | 0.02 | 70.20 | 11.8 | 25.92 | 10.12 | 29.09 |
| ODO1_MOUSE | Q60597 | 2-oxoglutarate dehydrogenase E1 component, mitochondrial | ID106221 | 1.82 | 4 | 0.01 | 40.17 | 3.9 | 16.83 | 14.29 | 35.88 |
| ABCF3_MOUSE | Q8K268 | ATP-binding cassette sub-family F member 3 | ID106401 | 1.79 | 7 | 0.01 | 70.22 | 11.7 | 14.14 | 18.35 | 33.78 |
| GUAD_MOUSE | Q9R111 | Guanine deaminase | ID137693 | 1.78 | 12 | 0.04 | 120.22 | 26.9 | 32.03 | 5.63 | 33.72 |
| UBP5_MOUSE | P56399 | Ubiquitin carboxyl-terminal hydrolase 5 | ID371765 | 1.75 | 7 | 0.01 | 70.21 | 10 | 10.90 | 20.54 | 34.28 |
| NDRG2_MOUSE | Q9QYG0 | Protein NDRG2 | ID106678 | 1.70 | 4 | 0.02 | | 0.16 | 12.85 | 11.02 | 26.21 |
| PSB3_MOUSE | Q9R1P1 | Proteasome subunit beta type-3 | ID106979 | 1.70 | 5 | 0.04 | 50.23 | 22.4 | 23.86 | 9.34 | 24.47 |
| TRI72_MOUSE | Q1XH17 | Tripartite motif-containing protein | ID106550 | 1.68 | 15 | 0.00 | 150.24 | 28.9 | 7.80 | 19.64 | 31.74 |
| PYGB_MOUSE | Q8CI94 | Glycogen phosphorylase, brain forı | ID106268 | 1.64 | 8 | 0.00 | | 0.077 | 7.86 | 9.71 | 25.64 |
| CHM4B_MOUSE | Q9D8B3 | Charged multivesicular body protein 4b | ID236029 | 1.64 | 5 | 0.01 | 50.22 | 20.1 | 20.75 | 20.13 | 32.65 |
| MOES_MOUSE | P26041 | Moesin | ID653997 | 1.63 | 15 | 0.03 | 150.26 | 19.1 | 20.82 | 15.44 | 27.52 |
| COR1A_MOUSE | O89053 | Coronin-1A | ID120818 | 1.60 | 12 | 0.02 | 120.17 | 21 | 10.94 | 15.92 | 27.07 |
| COPD_MOUSE | Q5XJY5 | Coatomer subunit delta | ID106498 | 1.56 | 7 | 0.01 | 70.20 | 12.9 | 11.84 | 16.51 | 26.94 |
| LDHB_MOUSE | P16125 | L-lactate dehydrogenase B chain | ID171779 | 1.54 | 9 | 0.00 | 90.20 | 27.2 | 10.96 | 6.15 | 19.54 |
| NDRG2_MOUSE | Q9QYG0 | Protein NDRG2 | ID106712 | 1.54 | 4 | 0.01 | | 0.16 | 11.28 | 15.42 | 23.81 |
| GRP75_MOUSE | P38647 | Stress-70 protein, mitochondrial | ID133104 | 1.53 | 16 | 0.02 | 160.27 | 29.2 | 20.55 | 9.40 | 25.21 |
| NDRG2_MOUSE | Q9QYG0 | Protein NDRG2 | ID106675 | 1.52 | 3 | 0.01 | | 0.097 | 13.42 | 12.77 | 23.12 |
| ODO1_MOUSE | Q60597 | 2-oxoglutarate dehydrogenase E1 component, mitochondrial | ID106200 | 1.51 | 10 | 0.00 | | 0.12 | 12.70 | 8.82 | 21.82 |

**Proteins altered by $\leq$ 0.67 fold difference due to aging in mice at 4 months**

| | | | | | | | | | | | |
|---|---|---|---|---|---|---|---|---|---|---|---|
| ACY1_MOUSE | Q99JW2 | Aminoacylase-1 | ID106719 | 0.66 | 5 | 0.00 | 50.21 | 17.9 | 12.40 | 18.79 | 35.80 |
| MYH6_MOUSE | Q02566 | Myosin-6 | ID146761 | 0.65 | 34 | 0.00 | 340.29 | 18 | 9.46 | 10.76 | 27.78 |
| MYH6_MOUSE | Q02566 | Myosin-6 | ID106185 | 0.65 | 65 | 0.01 | 648.29 | 27.5 | 9.35 | 15.78 | 27.80 |
| PDK2_MOUSE | Q9JK42 | Pyruvate dehydrogenase [lipoamide] kinase isozyme 2, mitochondria | ID176784 | 0.65 | 8 | 0.01 | 78.26 | 25.3 | 13.54 | 12.11 | 27.34 |
| MYH6_MOUSE | Q02566 | Myosin-6 | ID129272 | 0.65 | 32 | 0.00 | 318.26 | 12.5 | 14.22 | 14.45 | 31.51 |
| MYH6_MOUSE | Q02566 | Myosin-6 | ID441410 | 0.64 | 34 | 0.01 | 338.30 | 15.5 | 18.30 | 17.68 | 32.89 |
| MYH6_MOUSE | Q02566 | Myosin-6 | ID106133 | 0.63 | 2 | 0.05 | 20.16 | 1.6 | 23.76 | 18.05 | 35.80 |
| MYH6_MOUSE | Q02566 | Myosin-6 | ID106123 | 0.63 | 54 | 0.02 | 540.28 | 28.2 | 8.47 | 23.86 | 31.21 |

| | | | | | | | | | | | |
|---|---|---|---|---|---|---|---|---|---|---|---|
| MYH6_MOUSE | Q02566 | Myosin-6 | ID440064 | 0.60 | 35 | 0.03 | 348.25 | 17.4 | 19.15 | 19.14 | 32.45 |
| ERF1_MOUSE | Q8BWY3 | Eukaryotic peptide chain release factor subunit 1 | ID106578 | 0.60 | 8 | 0.00 | 80.19 | 19.9 | 12.55 | 8.93 | 31.19 |
| TPM1_MOUSE | P58771 | Tropomyosin alpha-1 chain | ID106970 | 0.60 | 5 | 0.00 | 46.20 | 13.7 | 14.61 | 14.76 | 35.85 |
| MYH6_MOUSE | Q02566 | Myosin-6 | ID106506 | 0.59 | 30 | 0.00 | 296.32 | 12.6 | 19.02 | 16.85 | 38.31 |
| ILEUA_MOUSE | Q9D154 | Leukocyte elastase inhibitor A | ID106744 | 0.59 | 11 | 0.00 | 110.21 | 24.5 | 11.70 | 15.88 | 33.91 |
| MYH6_MOUSE | Q02566 | Myosin-6 | ID450800 | 0.59 | 36 | 0.03 | 358.29 | 15.8 | 19.45 | 29.01 | 37.37 |
| MYH6_MOUSE | Q02566 | Myosin-6 | ID449484 | 0.59 | 29 | 0.01 | 288.27 | 13.1 | 17.39 | 17.01 | 33.96 |
| CAP2_MOUSE | Q9CYT6 | Adenylyl cyclase-associated protei | ID122705 | 0.58 | 7 | 0.04 | 70.20 | 14.7 | 29.39 | 25.48 | 47.29 |
| ACTA_MOUSE | P62737 | Actin, aortic smooth muscle | ID106945 | 0.58 | 4 | 0.00 | 40.23 | 7.7 | 15.51 | 15.73 | 37.38 |
| MYH6_MOUSE | Q02566 | Myosin-6 | ID106501 | 0.57 | 31 | 0.01 | 306.27 | 14.3 | 25.76 | 18.32 | 44.97 |
| MYH6_MOUSE | Q02566 | Myosin-6 | ID128693 | 0.56 | 23 | 0.00 | 228.26 | 10.1 | 9.90 | 7.94 | 34.75 |
| MYH6_MOUSE | Q02566 | Myosin-6 | ID624196 | 0.56 | 3 | 0.01 | 30.15 | 1.5 | 21.29 | 25.52 | 41.07 |
| COQ9_MOUSE | Q8K1Z0 | Ubiquinone biosynthesis protein COQ9, mitochondrial | ID114165 | 0.55 | 3 | 0.02 | 30.18 | 8.3 | 25.31 | 9.89 | 42.75 |
| MYH6_MOUSE | Q02566 | Myosin-6 | ID472077 | 0.55 | 8 | 0.00 | 80.22 | 4.7 | 6.23 | 15.10 | 27.46 |
| MYH6_MOUSE | Q02566 | Myosin-6 | ID127885 | 0.54 | 24 | 0.00 | 240.30 | 11.8 | 17.21 | 16.82 | 39.19 |
| CH60_MOUSE | P63038 | 60kDa heat shock protein, mitochondrial | ID624781 | 0.54 | 12 | 0.02 | 120.26 | 22.9 | 27.40 | 7.87 | 47.92 |
| MYH6_MOUSE | Q02566 | Myosin-6 | ID333105 | 0.54 | 28 | 0.00 | 280.26 | 13.8 | 18.15 | 16.33 | 41.45 |
| MYH6_MOUSE | Q02566 | Myosin-6 | ID291087 | 0.53 | 13 | 0.02 | 130.17 | 5.9 | 23.01 | 17.45 | 40.94 |
| PSB4_MOUSE | P99026 | Proteasome subunit beta type-4 | ID283773 | 0.52 | 2 | 0.00 | 20.21 | 5.7 | 18.55 | 7.72 | 42.67 |
| MYH6_MOUSE | Q02566 | Myosin-6 | ID136652 | 0.50 | 19 | 0.01 | 190.29 | 9.9 | 20.03 | 21.79 | 40.91 |
| EF1D_MOUSE | P57776 | Elongation factor 1- delta | ID106771 | 0.50 | 7 | 0.00 | 70.20 | 22.1 | 25.78 | 21.98 | 50.66 |
| MYH6_MOUSE | Q02566 | Myosin-6 | ID326812 | 0.49 | 17 | 0.00 | 170.23 | 9.5 | 12.68 | 10.87 | 41.43 |
| MYH6_MOUSE | Q02566 | Myosin-6 | ID106440 | 0.49 | 10 | 0.03 | 100.30 | 5.6 | 15.57 | 34.01 | 42.16 |
| MYH6_MOUSE | Q02566 | Myosin-6 | ID106743 | 0.47 | 21 | 0.02 | 206.26 | 7.6 | 8.80 | 37.57 | 43.26 |
| ANXA6_MOUSE | P14824 | Annexin A6 | ID652661 | 0.47 | 25 | 0.01 | 250.28 | 34.5 | 21.87 | 18.36 | 44.74 |
| MYH6_MOUSE | Q02566 | Myosin-6 | ID106902 | 0.46 | 6 | 0.00 | 60.26 | 3.1 | 22.68 | 17.25 | 47.52 |
| ATPB_MOUSE | P56480 | ATP synthase subunit beta, mitochondrial | ID106799 | 0.46 | 11 | 0.00 | 110.27 | 17.6 | 20.63 | 9.98 | 49.60 |
| TALDO_MOUSE | Q93092 | Transaldolase | ID106797 | 0.46 | 5 | 0.01 | 50.16 | 16.9 | 19.12 | 10.50 | 43.64 |
| ACTA_MOUSE | P62737 | Actin, aortic smooth muscle | ID281409 | 0.45 | 4 | 0.00 | 40.16 | 11.9 | 13.99 | 13.82 | 44.59 |
| ATPB_MOUSE | P56480 | ATP synthase subunit beta, mitochondrial | ID106852 | 0.45 | 4 | 0.02 | 80.20 | 7.2 | 39.17 | 14.75 | 64.04 |

| | | | | | | | | | | | |
|---|---|---|---|---|---|---|---|---|---|---|---|
| MYH6_MOUSE | Q02566 | Myosin-6 | ID106752 | 0.45 | 7 | 0.00 | 70.23 | 4.2 | 15.98 | 19.63 | 48.72 |
| MYH6_MOUSE | Q02566 | Myosin-6 | ID348085 | 0.45 | 19 | 0.00 | 190.25 | 10.5 | 13.43 | 22.29 | 45.70 |
| PPME1_MOUSE | Q8BVQ5 | Protein phosphatase methyl esterase 1 | ID106704 | 0.44 | 9 | 0.01 | 90.20 | 17.1 | 26.92 | 25.83 | 51.19 |
| MYH6_MOUSE | Q02566 | Myosin-6 | ID106757 | 0.41 | 11 | 0.01 | 110.23 | 6.4 | 10.96 | 27.95 | 46.23 |
| MYH6_MOUSE | Q02566 | Myosin-6 | ID335701 | 0.40 | 5 | 0.01 | 50.22 | 2.9 | 22.97 | 18.65 | 50.86 |
| ATPB_MOUSE | P56480 | ATP synthase subunit beta, mitochondrial | ID254426 | 0.40 | 10 | 0.00 | 100.21 | 17.6 | 17.22 | 13.65 | 51.46 |
| MYH6_MOUSE | Q02566 | Myosin-6 | ID624192 | 0.31 | 5 | 0.01 | 50.17 | 2.8 | 91.23 | 34.48 | 138.87 |
| HNRPK_MOUSE | P61979 | Heterogeneous nuclear ribonucleoprotein K | ID106517 | 0.06 | 9 | 0.02 | 90.23 | 15.8 | 42.51 | 107.78 | 104.33 |

*Swissprot ID-Swissprot Entry name, Acc.No-Swissprot Accession number, *CV-Coefficient of variation
