# Supplementary material for: Proteomic analyses of age related changes in A.BY/SnJ mouse hearts
Source: Proteome Sci. 2013 Jul 1;11:29. doi: 10.1186/1477-5956-11-29 (PMC3704963; doi:10.1186/1477-5956-11-29)
Supplement: Additional file 2: Table S2 — List of protein identified with 2 peptide confidence by LC-MS/MS analysis. [file 1477-5956-11-29-S2.pdf]

Supplementary Table S2 . List of protein identified with 2peptide confidence by LC-MS/MS analysis

Swissprot ID	Protein Annotation	MW	Peptide Count	Sequence coverage
1433E_MOUSE	14-3-3 protein epsilon	29 kDa	6	31%
1433F_MOUSE	14-3-3 protein eta	28 kDa	2	10%
1433G_MOUSE	14-3-3 protein gamma	28 kDa	5	26%
1433T_MOUSE	14-3-3 protein theta	28 kDa	2	11%
1433Z_MOUSE	14-3-3 protein zeta/delta	28 kDa	3	13%
2AAA_MOUSE	Serine/threonine-protein phosphatase 2A 65 kDa regulatory subunit A alpha isoform	65 kDa	3	7%
3HIDH_MOUSE	3-hydroxyisobutyrate dehydrogenase, mitochondrial	35 kDa	4	19%
A1AT4_MOUSE	Alpha-1-antitrypsin 1-4	46 kDa	3	7%
A1AT5_MOUSE	Alpha-1-antitrypsin 1-5	46 kDa	4	9%
A2M_MOUSE	Alpha-2-macroglobulin	166 kDa	2	2%
AATC_MOUSE	Aspartate aminotransferase, cytoplasmic	46 kDa	9	26%
AATM_MOUSE	Aspartate aminotransferase, mitochondrial	47 kDa	11	26%
ACADL_MOUSE	Long-chain specific acyl-CoA dehydrogenase, mitochondrial	48 kDa	14	26%
ACADM_MOUSE	Medium-chain specific acyl-CoA dehydrogenase, mitochondrial	46 kDa	9	26%
ACADS_MOUSE	Short-chain specific acyl-CoA dehydrogenase, mitochondrial	45 kDa	7	17%
ACADV_MOUSE	Very long-chain specific acyl-CoA dehydrogenase, mitochondrial	71 kDa	15	26%
ACON_MOUSE	Aconitate hydratase, mitochondrial	85 kDa	24	38%
ACOT2_MOUSE	Acyl-coenzyme A thioesterase 2, mitochondrial	50 kDa	3	7%
ACPM_MOUSE	Acyl carrier protein, mitochondrial	17 kDa	2	12%
ACS2L_MOUSE	Acetyl-coenzyme A synthetase 2-like, mitochondrial	75 kDa	6	14%
ACSL1_MOUSE	Long-chain-fatty-acid--CoA ligase 1	78 kDa	11	18%
ACTC_MOUSE	Actin, alpha cardiac muscle 1	42 kDa	3	6%
ACTG_MOUSE	Actin, cytoplasmic 2	42 kDa	6	29%
ACTN2_MOUSE	Alpha-actinin-2	104 kDa	27	37%
ACTN4_MOUSE	Alpha-actinin-4	105 kDa	2	3%
ACTS_MOUSE	Actin, alpha skeletal muscle	42 kDa	12	51%
ADCK3_MOUSE	Chaperone activity of bc1 complex-like, mitochondrial	72 kDa	3	6%
ADT1_MOUSE	ADP/ATP translocase 1	33 kDa	12	43%
ADT2_MOUSE	ADP/ATP translocase 2	33 kDa	3	9%
AIFM1_MOUSE	Apoptosis-inducing factor 1, mitochondrial	67 kDa	6	12%
AL4A1_MOUSE	Delta-1-pyrroline-5-carboxylate dehydrogenase, mitochondrial	62 kDa	4	7%
ALBU_MOUSE	Serum albumin	69 kDa	32	59%
ALDH2_MOUSE	Aldehyde dehydrogenase, mitochondrial	57 kDa	3	8%
ALDOA_MOUSE	Fructose-bisphosphate aldolase A	39 kDa	13	47%
ALDR_MOUSE	Aldose reductase	36 kDa	4	11%
ANF_MOUSE	Atrial natriuretic factor	17 kDa	2	14%
ANXA2_MOUSE	Annexin A2	39 kDa	5	12%
ANXA5_MOUSE	Annexin A5	36 kDa	5	17%
ANXA6_MOUSE	Annexin A6	76 kDa	2	2%
AOFB_MOUSE	Amine oxidase [flavin-containing] B	59 kDa	2	6%
APOA1_MOUSE	Apolipoprotein A-I	31 kDa	4	23%
APOA4_MOUSE	Apolipoprotein A-IV	45 kDa	3	6%
APOC3_MOUSE	Apolipoprotein C-III	11 kDa	2	38%
APOOL_MOUSE	Apolipoprotein	29 kDa	2	10%
ARHL1_MOUSE	[Protein ADP-ribosylarginine] hydrolase-like protein 1	40 kDa	3	13%
AT1A1_MOUSE	Sodium/potassium-transporting ATPase subunit alpha-1	113 kDa	12	13%
AT1B1_MOUSE	Sodium/potassium-transporting ATPase subunit beta-1	35 kDa	3	12%
AT2A2_MOUSE	Sarcoplasmic/endoplasmic reticulum calcium ATPase 2	115 kDa	28	27%
AT5F1_MOUSE	ATP synthase subunit b, mitochondrial	29 kDa	5	15%
ATP5H_MOUSE	ATP synthase subunit d, mitochondrial	19 kDa	11	66%
ATP5J_MOUSE	ATP synthase-coupling factor 6, mitochondrial	12 kDa	5	40%
ATP5L_MOUSE	ATP synthase subunit g, mitochondrial	11 kDa	4	42%
ATPA_MOUSE	ATP synthase subunit alpha, mitochondrial	60 kDa	22	50%
ATPB_MOUSE	ATP synthase subunit beta, mitochondrial	56 kDa	25	66%
ATPD_MOUSE	ATP synthase subunit delta, mitochondrial	18 kDa	2	14%
ATPG_MOUSE	ATP synthase subunit gamma, mitochondrial	33 kDa	4	15%
ATPK_MOUSE	ATP synthase subunit f, mitochondrial	10 kDa	2	26%

ATPO_MOUSE	ATP synthase subunit	23 kDa	7	50%
BAG3_MOUSE	BAG family molecular chaperone regulator 3	62 kDa	2	6%
BASI_MOUSE	Basigin	42 kDa	3	10%
BDH_MOUSE	D-beta-hydroxybutyrate dehydrogenase, mitochondrial	38 kDa	4	18%
BR44_MOUSE	Brain protein 44	14 kDa	2	19%
CACP_MOUSE	Carnitine	71 kDa	6	15%
CAD13_MOUSE	Cadherin-13	78 kDa	2	4%
CALM_MOUSE	Calmodulin	17 kDa	4	51%
CALX_MOUSE	Calnexin	67 kDa	2	5%
CASQ2_MOUSE	Calsequestrin-2	48 kDa	5	22%
CATD_MOUSE	Cathepsin D	45 kDa	3	11%
CAZA2_MOUSE	F-actin-capping protein subunit alpha-2	33 kDa	2	9%
CD36_MOUSE	Platelet glycoprotein 4	53 kDa	2	6%
CH10_MOUSE	10 kDa heat shock protein, mitochondrial	11 kDa	3	27%
CH60_MOUSE	60 kDa heat shock protein, mitochondrial	61 kDa	15	33%
CHCH3_MOUSE	Coiled-coil-helix-coiled-coil-helix domain-containing protein 3, mitochondrial	26 kDa	3	8%
CISD1_MOUSE	CDGSH iron sulfur domain-containing protein 1	12 kDa	2	20%
CISY_MOUSE	Citrate synthase, mitochondrial	52 kDa	10	29%
CLH_MOUSE	Clathrin heavy chain 1	192 kDa	2	2%
CMC1_MOUSE	Calcium-binding mitochondrial carrier protein Aralar1	75 kDa	8	19%
CMC2_MOUSE	Calcium-binding mitochondrial carrier protein Aralar2	74 kDa	5	13%
CMYA5_MOUSE	Cardiomyopathy-associated protein 5	413 kDa	2	1%
CO1A1_MOUSE	Collagen alpha-1(I) chain	138 kDa	2	2%
CO1A2_MOUSE	Collagen alpha-2(I) chain	130 kDa	4	3%
CO3_MOUSE	Complement C3	186 kDa	5	4%
CO6A1_MOUSE	Collagen alpha-1(VI) chain	108 kDa	4	5%
COF2_MOUSE	Cofilin-2	19 kDa	3	19%
COQ9_MOUSE	Ubiquinone biosynthesis protein C	35 kDa	4	13%
COX2_MOUSE	Cytochrome c oxidase subunit 2	26 kDa	4	25%
COX41_MOUSE	Cytochrome c oxidase subunit 4 isoform 1, mitochondrial	20 kDa	6	37%
COX5A_MOUSE	Cytochrome c oxidase subunit 5A, mitochondrial	16 kDa	5	43%
COX5B_MOUSE	Cytochrome c oxidase subunit 5B, mitochondrial	14 kDa	4	38%
COX6C_MOUSE	Cytochrome c oxidase subunit 6C	8 kDa	3	26%
COX7R_MOUSE	Cytochrome c oxidase subunit 7A-related protein, mitochondrial	12 kDa	2	41%
CPT1B_MOUSE	Carnitine	88 kDa	6	8%
CPT2_MOUSE	Carnitine	74 kDa	6	14%
CRIP2_MOUSE	Cysteine-rich protein 2	23 kDa	4	34%
CRYAB_MOUSE	Alpha-crystallin B chain	20 kDa	3	18%
CSRP3_MOUSE	Cysteine and glycine-rich protein 3	21 kDa	5	28%
CTNA1_MOUSE	Catenin alpha-1	100 kDa	3	6%
CX6B1_MOUSE	Cytochrome c oxidase subunit 6B1	10 kDa	6	53%
CX7A1_MOUSE	Cytochrome c oxidase polypeptide 7A1, mitochondrial	9 kDa	2	29%
CX7A2_MOUSE	Cytochrome c oxidase polypeptide 7A2, mitochondrial	9 kDa	2	28%
CY1_MOUSE	Cytochrome c1, heme protein, mitochondrial	35 kDa	5	27%
CYC_MOUSE	Cytochrome c, somatic	12 kDa	5	49%
D3D2_MOUSE	3,2-trans-enoyl-CoA isomerase, mitochondrial	32 kDa	5	21%
DECR_MOUSE	2,4-dienoyl-CoA reductase, mitochondrial	36 kDa	3	8%
DESM_MOUSE	Desmin	53 kDa	23	51%
DHB8_MOUSE	Estradiol 17-beta-dehydrogenase 8	27 kDa	2	10%
DHE3_MOUSE	Glutamate dehydrogenase 1, mitochondrial	61 kDa	3	9%
DHSA_MOUSE	Succinate dehydrogenase [ubiquinone] flavoprotein subunit, mitochondrial	73 kDa	15	33%
DHSB_MOUSE	Succinate dehydrogenase [ubiquinone] iron-sulfur subunit, mitochondrial	32 kDa	8	34%
DLDH_MOUSE	Dihydrolipoyl dehydrogenase, mitochondrial	54 kDa	8	23%
DMD_MOUSE	Dystrophin	426 kDa	4	1%
DOPD_MOUSE	D-dopachrome decarboxylase	13 kDa	2	17%
DPYL2_MOUSE	Dihydropyrimidinase-related protein 2	62 kDa	4	6%
ECH1_MOUSE	Delta(3,5)-Delta(2,4)-dienoyl-CoA isomerase, mitochondrial	36 kDa	4	20%
ECHA_MOUSE	Trifunctional enzyme subunit alpha, mitochondrial	83 kDa	31	45%
ECHB_MOUSE	Trifunctional enzyme subunit beta, mitochondrial	51 kDa	10	16%
ECHM_MOUSE	Enoyl-CoA hydratase, mitochondrial	31 kDa	6	28%

EF1A2_MOUSE	Elongation factor 1-alpha 2	50 kDa	6	18%
EF1B_MOUSE	Elongation factor 1-beta	25 kDa	2	12%
EF2_MOUSE	Elongation factor 2	95 kDa	8	12%
EFTU_MOUSE	Elongation factor Tu, mitochondrial	50 kDa	6	20%
EHD4_MOUSE	EH domain-containing protein 4	61 kDa	6	9%
ENOA_MOUSE	Alpha-enolase	47 kDa	5	17%
ENOB_MOUSE	Beta-enolase	47 kDa	12	27%
ES1_MOUSE	ES1 protein homolog, mitochondrial	28 kDa	3	14%
ESTD_MOUSE	S-formylglutathione hydrolase	31 kDa	2	15%
ESTN_MOUSE	Liver carboxylesterase N	61 kDa	5	10%
ETFA_MOUSE	Electron transfer flavoprotein subunit alpha, mitochondrial	35 kDa	13	48%
ETFB_MOUSE	Electron transfer flavoprotein subunit beta	28 kDa	8	39%
ETFD_MOUSE	Electron transfer flavoprotein-ubiquinone oxidoreductase, mitochondrial	68 kDa	11	20%
FABP4_MOUSE	Fatty acid-binding protein, adipocyte	15 kDa	3	20%
FABPH_MOUSE	Fatty acid-binding protein, heart	15 kDa	3	28%
FAHD1_MOUSE	Fumarylacetoacetate hydrolase domain-containing protein 1	25 kDa	2	14%
FETUA_MOUSE	Alpha-2-HS-glycoprotein	37 kDa	4	20%
FHL2_MOUSE	Four and a half LIM domains protein 2	32 kDa	7	31%
FIBB_MOUSE	Fibrinogen beta chain	55 kDa	5	9%
FLNA_MOUSE	Filamin-A	281 kDa	4	3%
FLNC_MOUSE	Filamin-C	291 kDa	8	3%
FUMH_MOUSE	Fumarate hydratase, mitochondrial	54 kDa	9	25%
G3P_MOUSE	Glyceraldehyde-3-phosphate dehydrogenase	36 kDa	9	44%
G6PI_MOUSE	Glucose-6-phosphate isomerase	63 kDa	6	13%
GDIB_MOUSE	Rab GDP dissociation inhibitor beta	51 kDa	4	11%
GLYG_MOUSE	Glycogenin-1	37 kDa	2	10%
GRP75_MOUSE	Stress-70 protein, mitochondrial	74 kDa	14	22%
GRP78_MOUSE	78 kDa glucose-regulated protein	72 kDa	7	10%
GSTK1_MOUSE	Glutathione S-transferase kappa 1	26 kDa	3	16%
GSTM1_MOUSE	Glutathione S-transferase Mu 1	26 kDa	5	29%
GSTM2_MOUSE	Glutathione S-transferase Mu 2	26 kDa	2	6%
GSTO1_MOUSE	Glutathione S-transferase omega-1	27 kDa	2	8%
GSTP1_MOUSE	Glutathione S-transferase P 1	24 kDa	2	15%
H2B1B_MOUSE	Histone H2B type 1-B	14 kDa	2	19%
H4_MOUSE	Histone H4	11 kDa	5	50%
HBA_MOUSE	Hemoglobin subunit alpha	15 kDa	6	43%
HBB1_MOUSE	Hemoglobin subunit beta-1	16 kDa	5	39%
HBB2_MOUSE	Hemoglobin subunit beta-2	16 kDa	11	73%
HCD2_MOUSE	3-hydroxyacyl-CoA dehydrogenase type-2	27 kDa	4	29%
HCDH_MOUSE	Hydroxyacyl-coenzyme A dehydrogenase, mitochondrial	34 kDa	7	39%
HEMO_MOUSE	Hemopexin	51 kDa	4	10%
HIBCH_MOUSE	3-hydroxyisobutyryl-CoA hydrolase, mitochondrial	43 kDa	7	19%
HINT1_MOUSE	Histidine triad nucleotide-binding protein 1	14 kDa	2	17%
HNRPK_MOUSE	Heterogeneous nuclear ribonucleoprotein K	51 kDa	2	6%
HS90A_MOUSE	Heat shock protein HSP 90-alpha	85 kDa	2	4%
HS90B_MOUSE	Heat shock protein HSP 90-beta	83 kDa	12	18%
HSDL2_MOUSE	Hydroxysteroid dehydrogenase-like protein 2	54 kDa	2	4%
HSP74_MOUSE	Heat shock 70 kDa protein 4	94 kDa	3	6%
HSP7C_MOUSE	Heat shock cognate 71 kDa protein	71 kDa	15	22%
HSPB1_MOUSE	Heat shock protein beta-1	23 kDa	4	28%
HXK1_MOUSE	Hexokinase-1	108 kDa	4	2%
HXK2_MOUSE	Hexokinase-2	103 kDa	2	3%
HYES_MOUSE	Epoxide hydrolase 2	63 kDa	5	14%
IDH3A_MOUSE	Isocitrate dehydrogenase [NAD] subunit alpha, mitochondrial	40 kDa	9	31%
IDH3G_MOUSE	Isocitrate dehydrogenase [NAD] subunit gamma, mitochondrial	43 kDa	2	13%
IDHP_MOUSE	Isocitrate dehydrogenase [NADP], mitochondrial	51 kDa	21	45%
IMMT_MOUSE	Mitochondrial inner membrane protein	84 kDa	13	17%
IVD_MOUSE	Isovaleryl-CoA dehydrogenase, mitochondrial	46 kDa	5	11%
K1881_MOUSE	Protein KIAA1881	139 kDa	3	3%
K6PF_MOUSE	6-phosphofructokinase, muscle type	85 kDa	6	10%
KAD1_MOUSE	Adenylate kinase isoenzyme 1	22 kDa	7	41%
KAD3_MOUSE	GTP:AMP phosphotransferase mitochondrial	25 kDa	2	7%

KCC2D_MOUSE	Calcium/calmodulin-dependent protein kinase type II delta chain	56 kDa	2	9%
KCRB_MOUSE	Creatine kinase B-type	43 kDa	2	5%
KCRM_MOUSE	Creatine kinase M-type	43 kDa	15	43%
KCRS_MOUSE	Creatine kinase, sarcomeric mitochondrial	47 kDa	18	53%
KCY_MOUSE	UMP-CMP kinase	22 kDa	2	11%
KPYM_MOUSE	Pyruvate kinase isozymes M1/M2	58 kDa	14	30%
LAMA2_MOUSE	Laminin subunit alpha-2	343 kDa	9	3%
LAMB1_MOUSE	Laminin subunit beta-1	197 kDa	5	3%
LAMB2_MOUSE	Laminin subunit beta-2	196 kDa	2	1%
LAMC1_MOUSE	Laminin subunit gamma-1	177 kDa	9	8%
LDB3_MOUSE	LIM domain-binding protein 3	76 kDa	14	24%
LDHA_MOUSE	L-lactate dehydrogenase A chain	36 kDa	11	37%
LDHB_MOUSE	L-lactate dehydrogenase B chain	37 kDa	12	36%
LGUL_MOUSE	Lactoylglutathione lyase	21 kDa	2	16%
LMNA_MOUSE	Lamin-A/C	74 kDa	4	4%
LMNB1_MOUSE	Lamin-B1	67 kDa	2	4%
LPPRC_MOUSE	Leucine-rich PPR motif-containing protein, mitochondrial	157 kDa	2	1%
LUM_MOUSE	Lumican	38 kDa	3	7%
M2OM_MOUSE	Mitochondrial 2-oxoglutarate/malate carrier protein	34 kDa	3	12%
MACD1_MOUSE	MACR	35 kDa	2	12%
MCCA_MOUSE	Methylcrotonoyl-CoA carboxylase subunit alpha, mitochondrial	79 kDa	4	4%
MCCB_MOUSE	Methylcrotonoyl-CoA carboxylase beta chain, mitochondrial	61 kDa	2	4%
MDHC_MOUSE	Malate dehydrogenase, cytoplasmic	37 kDa	9	41%
MDHM_MOUSE	Malate dehydrogenase, mitochondrial	36 kDa	17	60%
MIF_MOUSE	Macrophage migration inhibitory factor	13 kDa	2	18%
MLE3_MOUSE	Myosin light chain 3, skeletal muscle isoform	17 kDa	2	17%
MLRA_MOUSE	Myosin regulatory light chain 2, atrial isoform	19 kDa	6	54%
MLRV_MOUSE	Myosin regulatory light chain 2, ventricular/cardiac muscle isoform	19 kDa	14	81%
MMSA_MOUSE	Methylmalonate-semialdehyde dehydrogenase [acylating], mitochondrial	58 kDa	8	15%
MOES_MOUSE	Moesin	68 kDa	3	8%
MOT1_MOUSE	Monocarboxylate transporter 1	53 kDa	2	7%
MPCP_MOUSE	Phosphate carrier protein, mitochondrial	40 kDa	6	20%
MPI_MOUSE	Mannose-6-phosphate isomerase	47 kDa	3	7%
MUG1_MOUSE	Murinoglobulin-1	165 kDa	3	2%
MUTA_MOUSE	Methylmalonyl-CoA mutase, mitochondrial	83 kDa	2	6%
MYG_MOUSE	Myoglobin	17 kDa	9	61%
MYH11_MOUSE	Myosin-11	227 kDa	4	2%
MYH6_MOUSE	Myosin-6	224 kDa	144	58%
MYH9_MOUSE	Myosin-9	226 kDa	2	2%
MYL3_MOUSE	Myosin light chain 3	22 kDa	16	75%
MYL4_MOUSE	Myosin light chain 4	21 kDa	4	28%
MYL6_MOUSE	Myosin light polypeptide 6	17 kDa	2	17%
MYOM1_MOUSE	Myomesin-1	185 kDa	15	11%
MYOZ2_MOUSE	Myozenin-2	30 kDa	4	19%
MYPC3_MOUSE	Myosin-binding protein C, cardiac-type	141 kDa	30	27%
NACAM_MOUSE	Nascent polypeptide-associated complex subunit alpha, muscle-specific form	221 kDa	4	2%
NAMPT_MOUSE	Nicotinamide phosphoribosyltransferase	55 kDa	2	7%
NDKB_MOUSE	Nucleoside diphosphate kinase B	17 kDa	5	39%
NDRG2_MOUSE	Protein NDRG2	41 kDa	2	15%
NDUA2_MOUSE	NADH dehydrogenase [ubiquinone] 1 alpha subcomplex subunit 2	11 kDa	2	24%
NDUA4_MOUSE	NADH dehydrogenase [ubiquinone] 1 alpha subcomplex subunit 4	9 kDa	3	37%
NDUA5_MOUSE	NADH dehydrogenase [ubiquinone] 1 alpha subcomplex subunit 5	13 kDa	2	12%
NDUA7_MOUSE	NADH dehydrogenase [ubiquinone] 1 alpha subcomplex subunit 7	13 kDa	3	28%
NDUA8_MOUSE	NADH dehydrogenase [ubiquinone] 1 alpha subcomplex subunit 8	20 kDa	2	12%
NDUA9_MOUSE	NADH dehydrogenase [ubiquinone] 1 alpha subcomplex subunit 9, mitochondrial	43 kDa	3	14%
NDUAA_MOUSE	NADH dehydrogenase [ubiquinone] 1 alpha subcomplex subunit 10, mitochondrial	41 kDa	9	21%
NDUAC_MOUSE	NADH dehydrogenase [ubiquinone] 1 alpha subcomplex subunit 12	17 kDa	2	18%
NDUAD_MOUSE	NADH dehydrogenase [ubiquinone] 1 alpha subcomplex subunit 13	17 kDa	3	22%
NDUB3_MOUSE	NADH dehydrogenase [ubiquinone] 1 beta subcomplex subunit 3	12 kDa	2	17%

NDUB4_MOUSE	NADH dehydrogenase [ubiquinone] 1 beta subcomplex subunit 4	15 kDa	2	27%
NDUB8_MOUSE	NADH dehydrogenase [ubiquinone] 1 beta subcomplex subunit 8, mitochondrial	22 kDa	2	21%
NDUB9_MOUSE	NADH dehydrogenase [ubiquinone] 1 beta subcomplex subunit 9	22 kDa	2	27%
NDUBA_MOUSE	NADH dehydrogenase [ubiquinone] 1 beta subcomplex subunit 10	21 kDa	4	32%
NDUBB_MOUSE	NADH dehydrogenase [ubiquinone] 1 beta subcomplex subunit 11, mitochondrial	17 kDa	2	26%
NDUS1_MOUSE	NADH-ubiquinone oxidoreductase 75 kDa subunit, mitochondrial	80 kDa	21	33%
NDUS2_MOUSE	NADH dehydrogenase [ubiquinone] iron-sulfur protein 2, mitochondrial	53 kDa	7	17%
NDUS3_MOUSE	NADH dehydrogenase [ubiquinone] iron-sulfur protein 3, mitochondrial	30 kDa	6	30%
NDUS4_MOUSE	NADH dehydrogenase [ubiquinone] iron-sulfur protein 4, mitochondrial	20 kDa	3	20%
NDUS6_MOUSE	NADH dehydrogenase [ubiquinone] iron-sulfur protein 6, mitochondrial	13 kDa	2	20%
NDUS7_MOUSE	NADH dehydrogenase [ubiquinone] iron-sulfur protein 7, mitochondrial	25 kDa	3	12%
NDUS8_MOUSE	NADH dehydrogenase [ubiquinone] iron-sulfur protein 8, mitochondrial	24 kDa	2	14%
NDUV1_MOUSE	NADH dehydrogenase [ubiquinone] flavoprotein 1, mitochondrial	51 kDa	9	30%
NDUV2_MOUSE	NADH dehydrogenase [ubiquinone] flavoprotein 2, mitochondrial	27 kDa	3	16%
NID1_MOUSE	Nidogen-1	137 kDa	5	3%
NIPS2_MOUSE	Protein NipSnap homolog 2	33 kDa	4	14%
NNTM_MOUSE	NAD(P) transhydrogenase, mitochondrial	114 kDa	10	13%
OBSCN_MOUSE	Obscurin	966 kDa	3	0%
ODBA_MOUSE	2-oxoisovalerate dehydrogenase subunit alpha, mitochondrial	50 kDa	3	10%
ODO1_MOUSE	2-oxoglutarate dehydrogenase E1 component, mitochondrial	116 kDa	20	26%
ODO2_MOUSE	Dihydrolipoyllysine-residue succinyltransferase component of 2-oxoglutarate dehydrogenase complex, mitochondrial	49 kDa	4	12%
ODP2_MOUSE	Dihydrolipoyllysine-residue acetyltransferase component of pyruvate dehydrogenase complex, mitochondrial	68 kDa	4	10%
ODPA_MOUSE	Pyruvate dehydrogenase E1 component subunit alpha, somatic form, mitochondrial	43 kDa	13	41%
ODPB_MOUSE	Pyruvate dehydrogenase E1 component subunit beta, mitochondrial	39 kDa	9	34%
ODPX_MOUSE	Pyruvate dehydrogenase protein X component, mitochondrial	54 kDa	4	5%
OPA1_MOUSE	Dynamin-like 120 kDa protein, mitochondrial	111 kDa	3	4%
PARK7_MOUSE	Protein DJ-1	20 kDa	3	30%
PCCA_MOUSE	Propionyl-CoA carboxylase alpha chain, mitochondrial	80 kDa	4	5%
PCCB_MOUSE	Propionyl-CoA carboxylase beta chain, mitochondrial	58 kDa	4	8%
PDIA1_MOUSE	Protein disulfide-isomerase	57 kDa	2	6%
PDIA3_MOUSE	Protein disulfide-isomerase A3	57 kDa	4	11%
PEBP1_MOUSE	Phosphatidylethanolamine-binding protein 1	21 kDa	7	64%
PECI_MOUSE	Peroxisomal 3,2-trans-enoyl-CoA isomerase	39 kDa	2	7%
PGAM1_MOUSE	Phosphoglycerate mutase 1	29 kDa	2	12%
PGAM2_MOUSE	Phosphoglycerate mutase 2	29 kDa	6	21%
PGBM_MOUSE	Basement membrane-specific heparan sulfate proteoglycan core protein	398 kDa	9	2%
PGK1_MOUSE	Phosphoglycerate kinase 1	45 kDa	6	15%
PGM1_MOUSE	Phosphoglucomutase-1	62 kDa	7	18%
PHB_MOUSE	Prohibitin	30 kDa	3	10%
PHB2_MOUSE	Prohibitin-2	33 kDa	4	14%
PIMT_MOUSE	Protein-L-isoaspartate(D-aspartate)	25 kDa	2	20%
PLAK_MOUSE	Junction plakoglobin	82 kDa	4	7%
PP1B_MOUSE	Serine/threonine-protein phosphatase PP1-beta catalytic subunit	37 kDa	2	8%
PPIA_MOUSE	Peptidyl-prolyl cis-trans isomerase A	18 kDa	5	32%
PPIF_MOUSE	Peptidyl-prolyl cis-trans isomerase, mitochondrial	22 kDa	3	25%
PRDX1_MOUSE	Peroxiredoxin-1	22 kDa	5	25%
PRDX2_MOUSE	Peroxiredoxin-2	22 kDa	6	23%
PRDX3_MOUSE	Thioredoxin-dependent peroxide reductase, mitochondrial	28 kDa	2	5%
PRDX5_MOUSE	Peroxiredoxin-5, mitochondrial	22 kDa	6	40%
PRDX6_MOUSE	Peroxiredoxin-6	25 kDa	3	21%

PROF1_MOUSE	Profilin-1	15 kDa	3	21%
PTRF_MOUSE	Polymerase I and transcript release factor	44 kDa	5	17%
PUR8_MOUSE	Adenylosuccinate lyase	55 kDa	2	5%
PURA1_MOUSE	Adenylosuccinate synthetase isozyme 1	50 kDa	2	5%
PYGB_MOUSE	Glycogen phosphorylase, brain form	97 kDa	7	10%
PYGM_MOUSE	Glycogen phosphorylase, muscle form	97 kDa	16	23%
QCR1_MOUSE	Cytochrome b-c1 complex subunit 1, mitochondrial	53 kDa	14	32%
QCR2_MOUSE	Cytochrome b-c1 complex subunit 2, mitochondrial	48 kDa	12	34%
QCR6_MOUSE	Cytochrome b-c1 complex subunit 6, mitochondrial	10 kDa	2	35%
QCR7_MOUSE	Cytochrome b-c1 complex subunit 7	14 kDa	8	50%
QCR8_MOUSE	Cytochrome b-c1 complex subunit 8	10 kDa	2	27%
RLA2_MOUSE	60S acidic ribosomal protein P2	12 kDa	2	39%
ROA2_MOUSE	Heterogeneous nuclear ribonucleoproteins A2/B1	37 kDa	4	14%
ROA3_MOUSE	Heterogeneous nuclear ribonucleoprotein A3	40 kDa	2	8%
RS14_MOUSE	40S ribosomal protein S14	16 kDa	2	16%
RSSA_MOUSE	40S ribosomal protein SA	33 kDa	2	10%
S10A1_MOUSE	Protein S100-A1	11 kDa	2	23%
SAM50_MOUSE	Sorting and assembly machinery component 50 homolog	52 kDa	2	5%
SAP_MOUSE	Sulfated glycoprotein 1	61 kDa	2	5%
SBP1_MOUSE	Selenium-binding protein 1	53 kDa	6	15%
SCOT1_MOUSE	Succinyl-CoA:3-ketoacid-coenzyme A transferase 1, mitochondrial	56 kDa	11	34%
SDPR_MOUSE	Serum deprivation-response protein	47 kDa	3	7%
SERPH_MOUSE	Serpin H1	47 kDa	2	10%
SODC_MOUSE	Superoxide dismutase [Cu-Zn]	16 kDa	5	32%
SODM_MOUSE	Superoxide dismutase [Mn], mitochondrial	25 kDa	2	13%
SPA3K_MOUSE	Serine protease inhibitor A3K	47 kDa	4	16%
SPRE_MOUSE	Sepiapterin reductase	28 kDa	2	12%
SPTA2_MOUSE	Spectrin alpha chain, brain	285 kDa	12	5%
SPTB2_MOUSE	Spectrin beta chain, brain 1	274 kDa	12	4%
SRBS1_MOUSE	Sorbin and SH3 domain-containing protein 1	143 kDa	2	2%
SRBS2_MOUSE	Sorbin and SH3 domain-containing protein 2	132 kDa	3	2%
SRCA_MOUSE	Sarcalumenin	99 kDa	11	15%
SSDH_MOUSE	Succinate-semialdehyde dehydrogenase, mitochondrial	56 kDa	3	6%
SUCA_MOUSE	Succinyl-CoA ligase [GDP-forming] subunit alpha, mitochondrial	36 kDa	2	7%
SUCB1_MOUSE	Succinyl-CoA ligase [ADP-forming] subunit beta, mitochondrial	50 kDa	9	24%
SUCB2_MOUSE	Succinyl-CoA ligase [GDP-forming] subunit beta, mitochondrial	47 kDa	3	6%
TAGL_MOUSE	Transgelin	23 kDa	2	12%
TAGL2_MOUSE	Transgelin-2	22 kDa	3	15%
TALDO_MOUSE	Transaldolase	37 kDa	2	6%
TBA1C_MOUSE	Tubulin alpha-1C chain	50 kDa	3	11%
TBA4A_MOUSE	Tubulin alpha-4A chain	50 kDa	8	23%
TBB2C_MOUSE	Tubulin beta-2C chain	50 kDa	10	35%
TBB5_MOUSE	Tubulin beta-5 chain	50 kDa	2	6%
TERA_MOUSE	Transitional endoplasmic reticulum ATPase	89 kDa	11	13%
TGM2_MOUSE	Protein-glutamine gamma-glutamyltransferase 2	77 kDa	5	8%
THEM2_MOUSE	Thioesterase superfamily member 2	15 kDa	2	16%
THIL_MOUSE	Acetyl-CoA acetyltransferase, mitochondrial	45 kDa	10	37%
THIM_MOUSE	3-ketoacyl-CoA thiolase, mitochondrial	42 kDa	17	66%
TIM13_MOUSE	Mitochondrial import inner membrane translocase subunit Tim13	10 kDa	2	25%
TITIN_MOUSE	Titin	3906 kDa	306	10%
TMOD1_MOUSE	Tropomodulin-1	40 kDa	3	12%
TNNC1_MOUSE	Troponin C, slow skeletal and cardiac muscles	18 kDa	4	28%
TNNI3_MOUSE	Troponin I, cardiac muscle	24 kDa	8	28%
TNNT2_MOUSE	Troponin T, cardiac muscle	36 kDa	6	20%
TPIS_MOUSE	Triosephosphate isomerase	27 kDa	10	44%
TPM1_MOUSE	Tropomyosin alpha-1 chain	33 kDa	29	66%
TPM4_MOUSE	Tropomyosin alpha-4 chain	28 kDa	2	9%
TRFE_MOUSE	Serotransferrin	77 kDa	17	25%
TRI72_MOUSE	Tripartite motif-containing protein 72	53 kDa	5	10%
TTHY_MOUSE	Transthyretin	16 kDa	2	19%
UBA1_MOUSE	Ubiquitin-like modifier-activating enzyme 1	118 kDa	5	7%
UBE2N_MOUSE	Ubiquitin-conjugating enzyme E2 N	17 kDa	2	17%
UBIQ_MOUSE	Ubiquitin	9 kDa	2	33%

UCRI_MOUSE	Cytochrome b-c1 complex subunit Rieske, mitochondrial	29 kDa	6	30%
VDAC1_MOUSE	Voltage-dependent anion-selective channel protein 1	32 kDa	8	33%
VDAC2_MOUSE	Voltage-dependent anion-selective channel protein 2	32 kDa	6	26%
VDAC3_MOUSE	Voltage-dependent anion-selective channel protein 3	31 kDa	4	18%
VIME_MOUSE	Vimentin	54 kDa	13	25%
VINC_MOUSE	Vinculin	117 kDa	10	9%
