# Supplementary material for: Proteomic analyses of age related changes in A.BY/SnJ mouse hearts
Source: Proteome Sci. 2013 Jul 1;11:29. doi: 10.1186/1477-5956-11-29 (PMC3704963; doi:10.1186/1477-5956-11-29)
Supplement: Additional file 3: Table S3 — List of proteins showing altered levels in hearts of 4 and 6 months old mice compared to 1 month old mice detected by LC-MS/MS approach. [file 1477-5956-11-29-S3.pdf]

Table S3-Proteins displaying changes in 4 months old hearts compared to control mice detected by LC-MS/MS approach

Swissprot ID*	Protein Annotation	Acc.No*	MW	pI	Sub-cellular Location	Peptide Count	Sum of Norm. Spectra			Sequence Coverage	Fold Change	p-value	Fold Change	p-value	CV†	CV	CV
							Day 0	4 months	6 months		4 m vs Day 0	4 m vs Day 0	6 m vs Day 0	6 m vs Day 0	1 Month	4 Months	
BDH_MOUSE	D-beta-hydroxybut	Q80XN0	38 kDa	8.62	Mitochondrion matrix	4	2.00	22.88	46.72	18%	12.20	0.00	23.36	0.00	0.00	35.58	111.90
GDIB_MOUSE	Rab GDP dissociati	Q61598	51 kDa	5.93	Cytoplasm Membrane; Peripheral membrane	4	3.16	11.44	n.d	11%	3.86	0.01			73.42	22.54	75.97
ARHL1_MOUSE	[Protein ADP-ribos	Q8BGK2	40 kDa	5.62		3	4.58	15.19	9.56	13%	3.54	0.04	2.09	0.24	112.66	36.35	83.32
ODBA_MOUSE	2-oxoisovalerate d	P50136	50 kDa	6.05	Mitochondrion matrix	3	3.16	8.81	n.d.	10%	2.97	0.03			73.42	35.15	71.61
IDH3G_MOUSE	Isocitrate dehydrog	P70404	43 kDa	8.66	Mitochondrion	2	7.48	19.31	19.55	13%	2.75	0.03	2.61	0.09	74.15	30.69	67.35
NDUB9_MOUSE	NADH dehydrogen	Q9CQJ8	22 kDa	7.84	Mitochondrion inner membrane; Peripheral membrane protein	2	10.56	23.25	22.29	27%	2.35	0.08	2.11	0.09	100.92	16.76	65.87
CD36_MOUSE	Platelet glycoprote	Q08857	53 kDa	8.61	Membrane	2	12.41	26.44	28.38	6%	2.27	0.03	2.29	0.01	56.93	20.44	54.01
MYG_MOUSE	Myoglobin	P04247	17 kDa	7.23		9	186.77	374.06	581.01	61%	2.14	0.00	3.11	0.00	35.02	6.70	44.46
NDUA9_MOUSE	NADH dehydrogen	Q9DC69	43 kDa	9.48	Mitochondrion matrix	3	13.76	26.44	30.75	14%	2.05	0.10	2.23	0.03	80.55	20.44	57.61
COX6C_MOUSE	Cytochrome c oxid	Q9CPQ1	8 kDa	10.13	Mitochondrion inner membrane	3	9.74	18.56	18.20	26%	2.03	0.09	1.87	0.16	77.94	16.69	55.75
LAMA2_MOUSE	Laminin subunit al	Q60675	343 kDa	5.77	extracellular matrix; basement membrane	9	22.41	40.88	33.98	3%	1.95	0.05	1.52	0.38	63.26	4.20	48.15
CPT1B_MOUSE	Carnitine-O-palmit	Q924X2	88 kDa	8.65	Mitochondrion outer membrane	6	15.52	27.19	38.72	8%	1.87	0.05	2.49	0.00	19.92	34.56	44.59
ACTS_MOUSE	Actin, alpha skelet	P68134	42 kDa	5.23	Cytoplasm cytoskeleton	12	509.27	814.50	1021.78	51%	1.71	0.01	2.01	0.00	20.40	17.75	33.80
ACS2L_MOUSE	Acetyl-coenzyme A	Q99NB1	75 kDa	5.98	Mitochondrion matrix	6	16.54	25.50	20.81	14%	1.64	0.00	1.26	0.27	19.35	0.00	29.04
MMSA_MOUSE	Methylmalonate-s	Q9EQ20	58 kDa	7.02	Mitochondrion	8	24.51	37.31	29.17	15%	1.62	0.03	1.19	0.49	31.32	13.57	33.14
CPT2_MOUSE	Carnitine-O-palmit	P52825	74 kDa	7.95	Mitochondrion inner membrane; Peripheral membrane	6	18.99	28.13	35.91	14%	1.58	0.05	1.89	0.00	7.17	28.94	32.86
NDUBA_MOUSE	NADH dehydrogen	Q9DCS9	21 kDa	8.36	Mitochondrion inner membrane; Peripheral membrane	4	35.06	51.19	42.91	32%	1.56	0.06	1.22	0.22	20.34	25.21	32.37
MPCP_MOUSE	Phosphate carrier	Q8VEM8	40 kDa	9.11	Mitochondrion inner membrane	6	43.41	61.88	67.48	20%	1.52	0.01	1.55	0.00	21.02	7.35	26.32
ETFD_MOUSE	Electron transfer fl	Q921G7	68 kDa	6.47	Mitochondrion inner membrane	11	48.33	67.88	67.29	20%	1.50	0.01	1.39	0.01	13.81	10.66	24.57
CACP_MOUSE	Carnitine-O-acetyl	P47934	71 kDa	8.63	Endoplasmic reticulum Peroxisome Mitochondrion inner membrane; Peripheral membrane	6	30.44	42.19	54.56	15%	1.48	0.01	1.79	0.00	6.26	13.49	23.56
ACOT2_MOUSE	Acyl-coenzyme A t	Q9QYR9	50 kDa	6.14	Mitochondrion matrix	3	11.01	14.81	11.30	7%	1.44	0.08	1.03	0.91	33.04	6.58	28.16
NNTM_MOUSE	NAD(P) transhydro	Q61941	114 kDa	6.45	Mitochondrion inner membrane	10	58.14	78.19	83.02	13%	1.43	0.03	1.43	0.04	15.67	17.06	24.75
TERA_MOUSE	Transitional endop	Q01853	89 kDa	5.14	Cytoplasm, Nucleus	11	33.24	44.06	36.73	13%	1.41	0.10	1.10	0.60	28.10	17.55	28.04
ODO1_MOUSE	2-oxoglutarate deh	Q60597	116 kDa	6.05	Mitochondrion matrix	20	119.28	155.44	162.28	26%	1.39	0.05	1.36	0.02	21.62	13.34	23.97
HBA_MOUSE	Hemoglobin subun	P01942	15 kDa	8.08		6	384.52	494.06	428.87	43%	1.37	0.08	1.12	0.59	24.48	13.17	24.42
AT5F1_MOUSE	ATP synthase subu	Q9CQQ7	29 kDa	8.55	Mitochondrion inner membrane	5	28.21	36.19	36.88	15%	1.37	0.02	1.31	0.05	15.54	9.71	20.57
CMC1_MOUSE	Calcium-binding m	Q8BH59	75 kDa	8.43	Mitochondrion inner membrane	8	36.81	45.75	57.14	19%	1.33	0.05	1.55	0.01	16.68	12.78	20.35
PEBP1_MOUSE	Phosphatidylethan	P70296	21 kDa	5.19	Cytoplasm	7	52.04	63.38	48.79	64%	1.30	0.08	0.94	0.72	20.38	10.85	20.39
HBB1_MOUSE	Hemoglobin subun	P02088	16 kDa	7.26		5	201.95	237.00	236.24	39%	1.25	0.06	1.17	0.16	15.31	7.84	16.41
PGAM2_MOUSE	Phosphoglycerate	O70250	29 kDa	8.65		6	63.21	49.31	47.86	21%	0.83	0.06	0.76	0.01	7.83	12.20	13.00
DESM_MOUSE	Desmin	P31001	53 kDa	5.21	Cytoplasm	23	234.48	182.81	166.32	51%	0.83	0.03	0.71	0.00	8.11	7.64	12.16

ATPA_MOUSE	ATP synthase subu	Q03265	60 kDa	8.28	Mitochondrion inner membrane	22	872.14	676.88	751.09	50%	0.83	0.01	0.86	0.01	6.52	6.23	11.57
ATPD_MOUSE	ATP synthase subu	Q9D3D9	18 kDa	4.46	Mitochondrion inner membrane	2	39.39	30.38	27.66	14%	0.82	0.01	0.70	0.03	3.88	8.55	11.56
VDAC1_MOUSE	Voltage-dependen	Q60932	32 kDa	8.55	Mitochondrion outer membrane; Cell membrane	8	95.84	73.88	92.00	33%	0.82	0.04	0.96	0.66	10.35	2.64	13.05
NDUA7_MOUSE	NADH dehydrogen	Q9Z1P6	13 kDa	10.17	Mitochondrion inner membrane; Peripheral membrane protein	3	43.82	32.63	25.87	28%	0.79	0.01	0.59	0.00	7.96	1.99	13.59
IMMT_MOUSE	Mitochondrial inne	Q8CAQ8	84 kDa	6.18	Mitochondrion inner membrane	13	97.73	72.00	81.61	17%	0.79	0.07	0.84	0.08	12.08	15.65	17.56
NDUA4_MOUSE	NADH dehydrogen	Q62425	9 kDa	9.52	Mitochondrion inner membrane; Peripheral membrane protein	3	67.82	49.88	57.32	37%	0.78	0.05	0.85	0.10	10.65	15.35	16.99
ATPO_MOUSE	ATP synthase subu	Q9DB20	23 kDa	9.8	Mitochondrion inner membrane	7	110.04	80.06	89.64	50%	0.78	0.10	0.81	0.08	15.99	15.54	19.77
TNNT2_MOUSE	Troponin T, cardiac	P50752	36 kDa	4.98		6	110.80	76.69	85.96	20%	0.74	0.09	0.78	0.05	17.70	18.97	23.02
ATP5H_MOUSE	ATP synthase subu	Q9DCX2	19 kDa	5.53	Mitochondrion inner membrane	11	202.24	138.38	144.11	66%	0.73	0.09	0.71	0.04	20.71	8.99	23.65
ALDR_MOUSE	Aldose reductase	P45376	36 kDa	6.79	Cytoplasm	4	23.48	15.94	19.04	11%	0.72	0.03	0.81	0.09	12.95	16.17	21.13
TAGL_MOUSE	Transgelin	P37804	23 kDa	8.86	Cytoplasm	2	20.22	13.69	14.79	12%	0.72	0.06	0.73	0.04	18.56	12.33	23.25
CH60_MOUSE	60 kDa heat shock	P63038	61 kDa	5.35	Mitochondrion matrix	15	159.56	107.44	109.67	33%	0.72	0.06	0.69	0.03	17.55	14.36	23.20
EHD4_MOUSE	EH domain-contain	Q9EQP2	61 kDa	6.33	Early endosome membrane; Peripheral membrane	6	28.18	18.38	21.56	9%	0.70	0.02	0.77	0.05	15.18	3.53	22.48
CYC_MOUSE	Cytochrome c, som	P62897	12 kDa	9.61	Mitochondrion matrix	5	151.65	91.69	72.90	49%	0.64	0.02	0.48	0.00	18.07	1.97	27.00
PHB_MOUSE	Prohibitin	P67778	30 kDa	5.57	Mitochondrion inner membrane	3	24.53	14.81	13.98	10%	0.64	0.05	0.57	0.09	23.23	6.58	29.80
ODPB_MOUSE	Pyruvate dehydrog	Q9D051	39 kDa	5.39	Mitochondrion matrix	9	126.89	73.50	95.78	34%	0.62	0.01	0.75	0.07	14.89	19.01	28.65
ECH1_MOUSE	Delta(3,5)-Delta(2,	O35459	36 kDa	6.01	Mitochondrion Peroxisome	4	30.91	17.25	28.37	20%	0.60	0.07	0.92	0.66	28.91	9.96	36.25
COQ9_MOUSE	Ubiquinone biosyn	Q8K1Z0	35 kDa	4.93	Mitochondrion	4	31.72	17.44	21.69	13%	0.59	0.09	0.68	0.12	30.33	28.18	39.20
ANXA5_MOUSE	Annexin A5	P48036	36 kDa	4.83		5	27.45	14.81	9.64	17%	0.58	0.08	0.35	0.01	32.06	6.58	39.29
PGK1_MOUSE	Phosphoglycerate	P09411	45 kDa	8.02	Cytoplasm	6	49.78	22.88	44.22	15%	0.49	0.03	0.89	0.57	28.12	24.75	44.09
CO1A2_MOUSE	Collagen alpha-2(I)	Q01149	130 kDa	10.00	extracellular matrix	4	72.45	31.50	34.44	3%	0.46	0.06	0.48	0.05	35.05	27.28	50.11
CO1A1_MOUSE	Collagen alpha-1(I)	P11087	138 kDa	9.28	extracellular matrix	2	36.89	15.00	12.36	2%	0.43	0.01	0.34	0.00	20.74	37.00	46.07
CX7A1_MOUSE	Cytochrome c oxid	P56392	9 kDa	8.71	Mitochondrion inner membrane	2	33.80	12.94	32.43	29%	0.41	0.01	0.96	0.82	8.23	77.82	49.60
GRP78_MOUSE	78 kDa glucose-reg	P20029	72 kDa	5.01	Endoplasmic reticulum lumen; Melanosome	7	39.94	13.69	24.67	10%	0.37	0.08	0.62	0.23	48.90	12.33	66.62
NDUB4_MOUSE	NADH dehydrogen	Q9CQC7	15 kDa	9.89	Mitochondrion inner membrane	2	25.68	8.63	19.67	27%	0.36	0.01	0.77	0.13	9.41	78.26	53.12
LMNA_MOUSE	Lamin-A/C	P48678	74 kDa	6.54	Nucleus Nucleus envelope	4	41.68	12.00	22.35	4%	0.31	0.02	0.54	0.02	28.29	74.41	62.74
PDIA1_MOUSE	Protein disulfide-is	P09103	57 kDa	4.75	Endoplasmic reticulum lumen; Melanosome Cell membrane; Peripheral membrane	2	12.11	3.19	2.00	0.06	0.28	0.02	0.17	0.00	34.50	71.32	67.91
