# Supplementary material for: Proteomic analyses of age related changes in A.BY/SnJ mouse hearts
Source: Proteome Sci. 2013 Jul 1;11:29. doi: 10.1186/1477-5956-11-29 (PMC3704963; doi:10.1186/1477-5956-11-29)
Supplement: Additional file 4: Figure S1 — Validation of age dependent changes in the level of proteins by immunohistochemical staining. [file 1477-5956-11-29-S4.pdf]

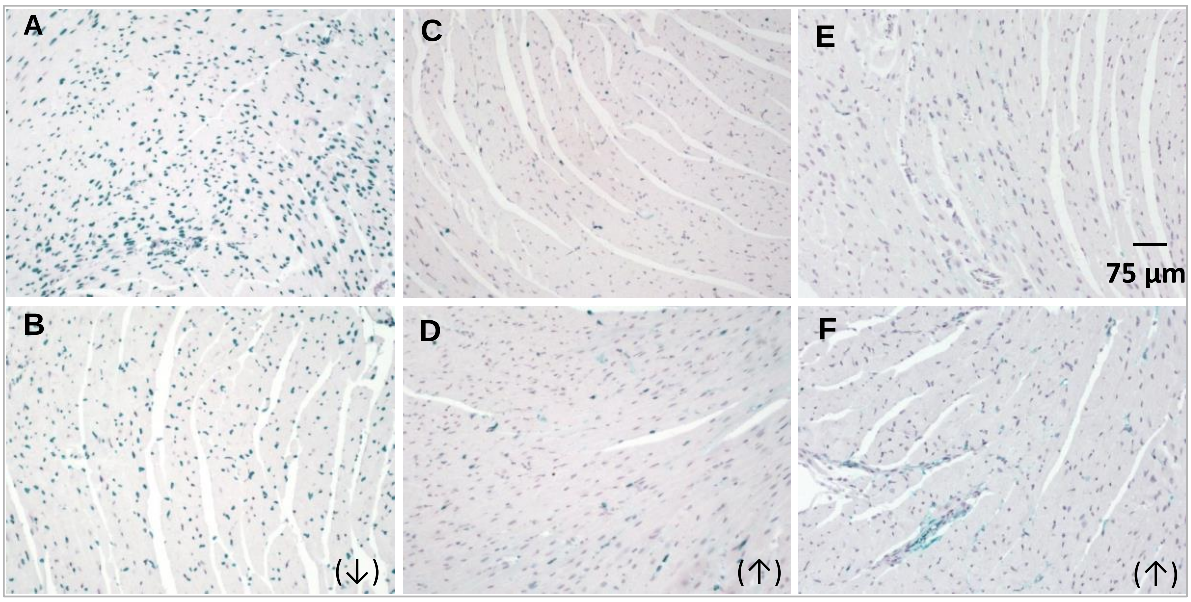


**Figure S2. Validation of age dependent changes in the level of proteins by immunohistochemical staining.** A, C, E represent immunohistochemical staining of heterogeneous nuclear ribonucleoprotein K (HNRPK), Rab GDP dissociation inhibitor beta (GDIB) and stress protein70 (GRP75) in 1 month old A.BY/SnJ mouse hearts. B, D, F- show immunohistochemical staining of the same proteins in 4 months old hearts. These data confirm decreased levels of HNRPK and increased levels of GDIB and GRP75 in 4 months old A.BY/SnJ mice hearts compared to juvenile mouse hearts.
