# Supplementary material for: Proteomic analyses of age related changes in A.BY/SnJ mouse hearts
Source: Proteome Sci. 2013 Jul 1;11:29. doi: 10.1186/1477-5956-11-29 (PMC3704963; doi:10.1186/1477-5956-11-29)
Supplement: Additional file 5: Figure S2 — Western Blot analysis results of ß-taxilin, CPT1B and CPT2. [file 1477-5956-11-29-S5.pdf]

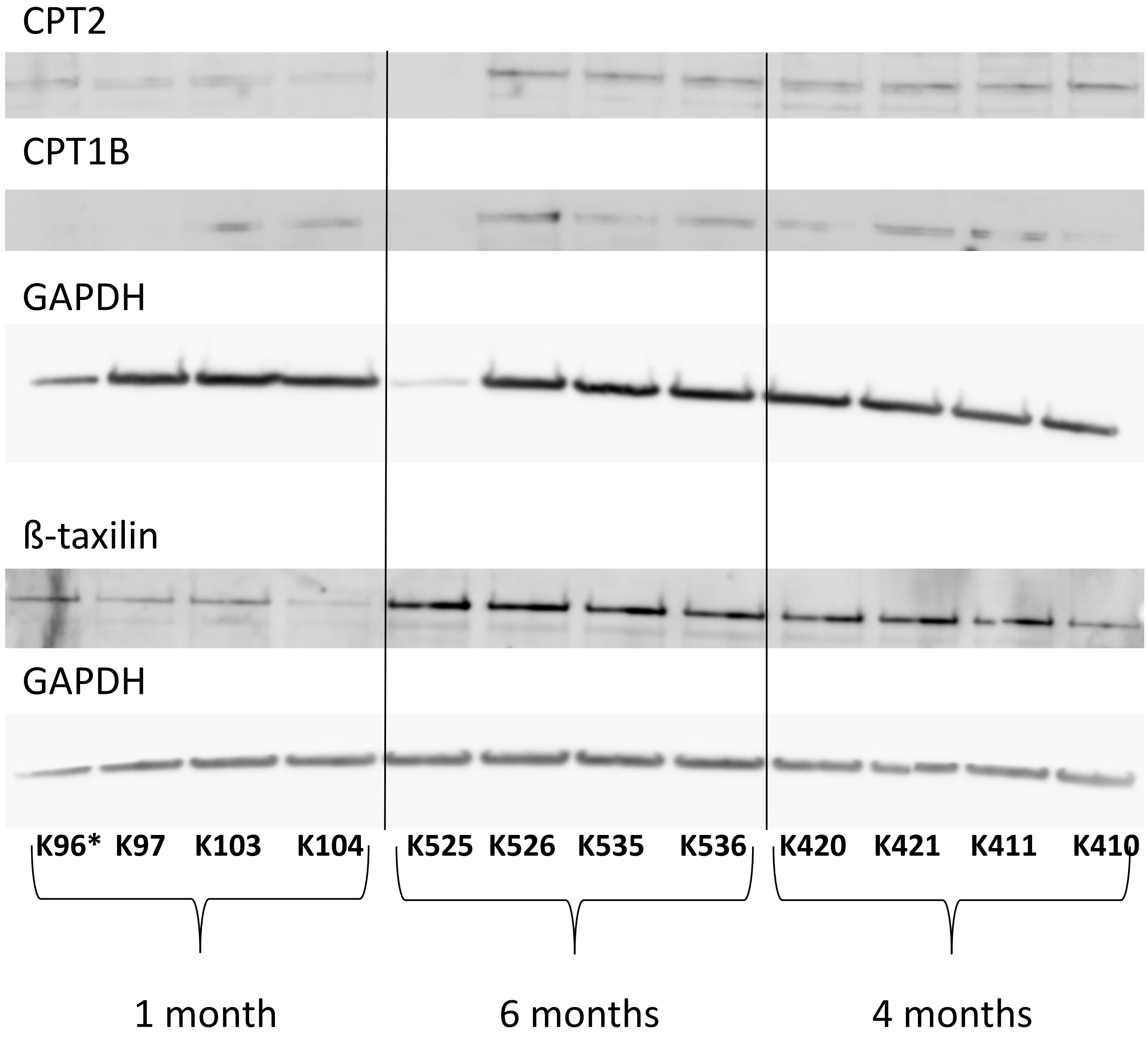

Figure S1 Western Blot analysis results of ß-taxilin, CPT1B and CPT2 . Signal intensities for GAPDH were recorded and used for normalization.

* Signal intensity of K96 was excluded from mean and p-value calculations for ß-taxilin due to background signal.
