# Supplementary material for: Proteomic analyses of age related changes in A.BY/SnJ mouse hearts
Source: Proteome Sci. 2013 Jul 1;11:29. doi: 10.1186/1477-5956-11-29 (PMC3704963; doi:10.1186/1477-5956-11-29)
Supplement: Additional file 6: Table S4 — Description of experiment settings for LC-MS/MS analysis and presentation of protein identification. [file 1477-5956-11-29-S6.pdf]

Table S4
Description of experiment settings for LC-MS/MS analysis and presentation of protein identification

1. LC-parameters and identification of protein spots from 2D gels

**LC-Parameters**	
LC gradient	0min-5%B-3m-5%-23m-35%-28m-60%-30m-100%-32m-100%-35m-0%
**Parameter**	**Settings**
Name of peaklist-generating software and release version (number or date)	extractMsn
Name of the search engine and release version (number or date)	Sequest 2.7 in Bioworks 3.3
Enzyme specificity considered	Fully tryptic
# of missed cleavages permitted	Missed cleavages=2
Fixed modification(s) (including residue specificity)	Carbamidomethylation at cysteine
Variable modification(s) (including residue specificity)	oxidation on methionine
Mass tolerance for precursor ions	10 ppm
Mass tolerance for fragment ions	1 Da
Name of database searched and release version/date	SwissProt database release 57.1 limited to mouse entries
Threshold score for accepting protein identification	at least two peptides passing the filter criteria mentioned below, keratins from keratinocytes and stratified epithelium were excluded from identification list
Threshold score/E-value for accepting *individual* MS/MS Spectra	charge dependent Xcorrelation score for two-fold charged peptides >2.2; three and four-fold charged peptides >3.70, RSp value =4; ion percent >60%.
Software/method used to evaluate site assignment	No PTM reported

2. Presentation of protein identification results for spots from 2D gels

**Information requested**	**Reported**
Accession number	SProt numbers and Entry name
Number of *unique* (in terms of amino acid sequence) peptides identified	Number of unique peptides is reported in Supplementary table S2
% sequence coverage identified from MS/MS data or a list of sequences identified	Percent sequence coverage is reported in Supplementary table S2
Additional information, such as a protein's name, protein score etc. may be included.	Name and protein score are shown additionally in Supplementary table S2
Single Peptide Protein IDs and PTMs<br>Include table with sequence identified<br>• The precursor m/z and charge<br>• Score / E-value for this peptide<br>Include MS/MS spectrum appropriately labeled, with masses detected as well as fragment assignments	Not reported in this manuscript

3. LC-parameters and identification of proteins in gel free LC-MS/MS analysis

**LC-Parameters**	
LC gradient	0min-0%B-15m-0%-27m5-30%-290m-60%-291m-100%-295m-100%-300m-0%
**Parameter**	**Settings**
Name of peaklist-generating software and release version (number or date)	ReadW in Sorcerer built 4.04 (SageN) with default parameters
Name of the search engine and release version (number or date)	Sequest 2.7 in Sorcerer built 4.04 (SageN)
Enzyme specificity considered	Fully tryptic
# of missed cleavages permitted	Missed clevages=2
Fixed modification(s) (including residue specificity)	Carbamidomethylation at cystein
Variable modification(s) (including residue specificity)	oxidation on methionine
Mass tolerance for precursor ions	10 ppm
Mass tolerance for fragment ions	1 Da
Name of database searched and release version/date	SwissProt database release 57.1 limited to mouse entries (forward-reverse)
Threshold score for accepting protein identification	Two significant peptides, protein probability >90 %
Threshold score/E-value for accepting *individual* MS/MS Spectra	Xcorrelation score for twofold charged peptides: 2.2; three- and four fold charged peptides: > 3.7; Delta Cn: 0.1
Software/method used to evaluate site assignment	No PTM reported

4. Mass spec parameters for gel free LC-MS/MS analysis

**MS Parameters**	
Survey full scan MS	m/z 300-2000
Resolution (R)	60,000 at m/z 400
Target Value	1,000,000 ions
Electrospray Voltage	2kV, no sheath and auxiliary gas flow
**MS/MS parameters**	
Ion selection	Top 5 intense ions in ion trap
CID target value	10,000 ions
Target ions dynamic exclusion	30s
Ion selection threshold	1000 counts
Activation Q value	0.25
Activation time	30ms
